# Elevated CO_2_ concentration promotes photosynthesis of grape (*Vitis vinifera* L. cv. ‘Pinot noir’) plantlet in vitro by regulating RbcS and Rca revealed by proteomic and transcriptomic profiles

**DOI:** 10.1186/s12870-019-1644-y

**Published:** 2019-01-29

**Authors:** Xin Zhao, Wen-Fang Li, Ying Wang, Zong-Huan Ma, Shi-Jin Yang, Qi Zhou, Juan Mao, Bai-Hong Chen

**Affiliations:** College of Horticulture, Gansu Agricultural University, Lanzhou, 730070 People’s Republic of China

**Keywords:** *Vitis vinifera*, iTRAQ labeling, RNA-Seq, Elevated CO_2_ concentration, Photosynthesis, Photoautotrophic

## Abstract

**Background:**

Plant photosynthesis can be improved by elevated CO_2_ concentration (eCO_2_). In vitro growth under CO_2_ enriched environment can lead to greater biomass accumulation than the conventional in micropropagation. However, little is know about how eCO_2_ promotes transformation of grape plantlets in vitro from heterotrophic to autotrophic. In addition, how photosynthesis-related genes and their proteins are expressed under eCO_2_ and the mechanisms of how eCO_2_ regulates *RbcS*, *Rca* and their proteins have not been reported.

**Results:**

Grape (*Vitis vinifera* L. cv. ‘Pinot Noir’) plantlets in vitro were cultured with 2% sucrose designated as control (CK), with eCO_2_ (1000 μmol·mol^− 1^) as C0, with both 2% sucrose and eCO_2_ as Cs. Here, transcriptomic and proteomic profiles associated with photosynthesis and growth in leaves of *V. vinifera* at different CO_2_ concentration were analyzed. A total of 1814 genes (465 up-regulated and 1349 down-regulated) and 172 proteins (80 up-regulated and 97 down-regulated) were significantly differentially expressed in eCO_2_ compared to CK. Photosynthesis-antenna, photosynthesis and metabolism pathways were enriched based on GO and KEGG. Simultaneously, 9, 6 and 48 proteins were involved in the three pathways, respectively. The leaf area, plantlet height, qP, ΦPSII and ETR increased under eCO_2_, whereas Fv/Fm and NPQ decreased. Changes of these physiological indexes are related to the function of DEPs. After combined analysis of proteomic and transcriptomic, the results make clear that eCO_2_ have different effects on gene transcription and translation. RbcS was not correlated with its mRNA level, suggesting that the change in the amount of RbcS is regulated at their transcript levels by eCO_2_. However, Rca was negatively correlated with its mRNA level, it is suggested that the change in the amount of its corresponding protein is regulated at their translation levels by eCO_2_.

**Conclusions:**

Transcriptomic, proteomic and physiological analysis were used to evaluate eCO2 effects on photosynthesis. The eCO_2_ triggered the RbcS and Rca up-regulated, thus promoting photosynthesis and then advancing transformation of grape plantlets from heterotrophic to autotrophic. This research will helpful to understand the influence of eCO_2_ on plant growth and promote reveal the mechanism of plant transformation from heterotrophic to autotrophic.

**Electronic supplementary material:**

The online version of this article (10.1186/s12870-019-1644-y) contains supplementary material, which is available to authorized users.

## Background

Increasing atmospheric CO_2_ concentration influences plant growth [1, 2]. Photosynthesis, respiration and water relations are the three primary physiological processes influenced by elevated CO_2_ concentration (eCO_2_) in plants [3]. CO_2_ concentration inside the culture vessels decreased when plantlets grown in vitro, which limits the photosynthetic rate of the plants [4, 5]. The biomass accumulation of the in vitro cultured plants increased under photoautotrophic and CO_2_ enrichment conditions, also affected nutrient absorption and secondary metabolism [6, 7].

Plantlet grown vigorously under CO_2_ enriched photoautotrophic and photomixotrophic conditions, with high photosynthetic photon flux density [8]. Photosynthetic response to light and CO_2_ increased with Rubisco activities and proteins of plantlets grown in vitro [7]. Rubisco, the main catalytic enzyme determines photosynthetic rate [8], would respond to eCO_2_ [9] and increase carboxylation efficiency under eCO_2_ [10]. Succinctly, the synthesis of the Rubisco holoenzyme is mainly affected by ribulose bisphosphate carboxylase small chain (RbcS) [11]. The activity of Rubisco is related to Rubisco activase (Rca) and other proteins [12, 13].

In some species, it is reported that the transcript levels of *RbcS* are differentially regulated by red and blue light or growth temperature [14]. The abundance of the *RbcS* multigene family transcript has been researched in many plants [15]. RbcS regulates Rubisco through coordinated expression of *RbcL* and RbcS in plants [11]. In addition to the folded RbcL subunits assemble [16], RbcS could combine more CO_2_ than the RbcL in all Rubiscos [17]. The detailed mechanism of RbcS mediated assembly of RbcL under different environment and how the expression of *RbcS* and its protein responds to eCO_2_ remains to be investigated. The Rca could gain energy from ATP hydrolysis to remodel Rubisco inhibitors and activate Rubisco [18]. Inhibit expression of Rca in some plants results in severe photoautotrophic growth defects [19]. Rca proteins belong to a subgroup of the ATPases associated with various cellular activities (AAA) called AAA+ [20]. There are two Rca forms both can activate Rubisco [21]. Rca is regulated by the intracellular ATP/ADP ratio [22] or the C-terminal extension of the α-isoform of Rca in some plants [18]. Some research indicated that Rca could reduce the effects of abiotic stresses on plants, such as high temperature, drought, salt [23–25] and heavy metal [26]. The expression of *Rca* is regulated by trans-acting factors in soybean [27]. The actual change mechanism of *Rca* expression and whether Rca related to other proteins under eCO_2_ is less studied.

‘Pinot Noir’ is a wine grape variety widely planted in worldwide and its growth influenced by various environmental factors [28]. The increasing CO_2_ concentration could promote plant growth. Although, many studies have focused on the effects of CO_2_ on grape ripening [29] and postharvest [30]. It is unclear the mechanism of how eCO_2_ affects the plant growth and photosynthesis. Additionally, there are a few reports on the analysis of transcriptome combined with proteome to study the effects of eCO_2_ on grape growth and development. In light of this situation, the experiment was conducted based on the hypothesis that eCO_2_ will enhance photosynthesis by regulating the expression of related genes and proteins in grape plantlets. Therefore, grape plantlets grown in vitro cultured with eCO_2_ were used in this study based on transcriptome, proteome and photosynthetic physiology analysis.

## Results

### Effects of eCO_2_ on growth and chlorophyll fluorescence

**Fig. 1:**
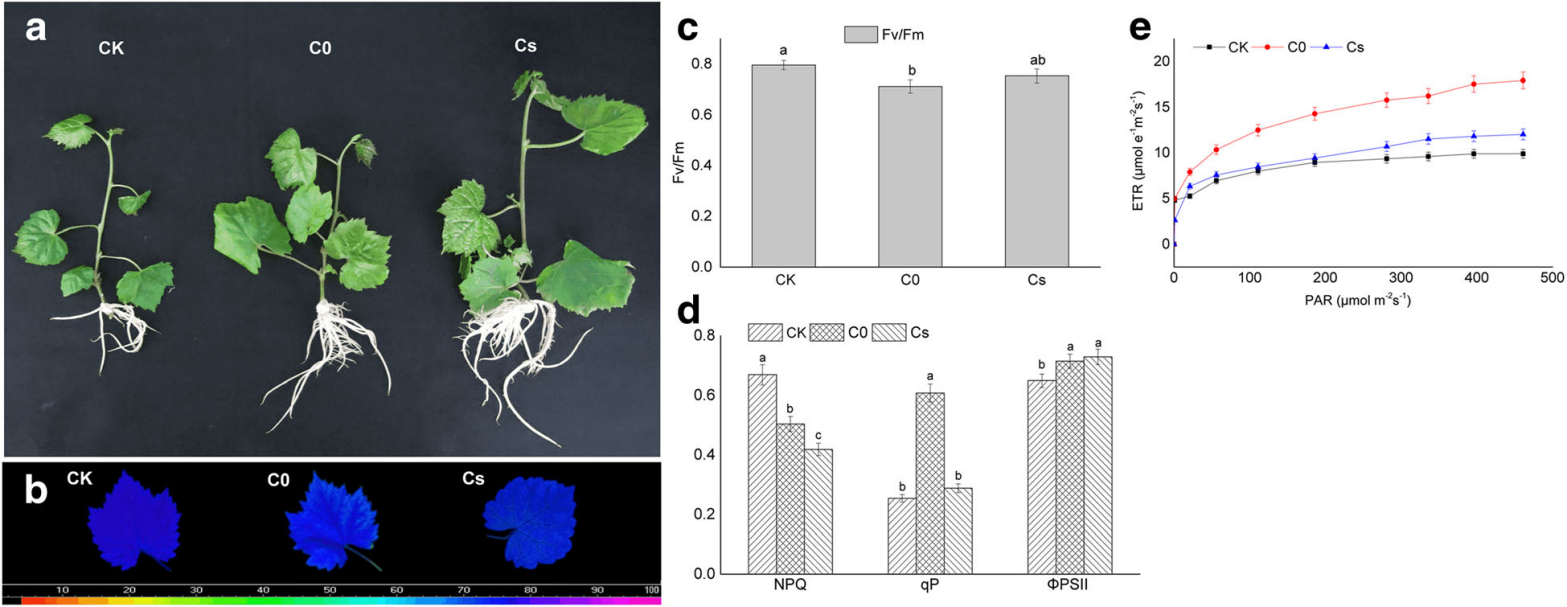
Phenotypical characteristics and fluorescence parameter changes of ‘Pinot Noir’ plantlet in vitro caused by eCO_2_ (1000 μmol·mol^− 1^) on the 25th day. **a** Changes of the morphology and growth. **b**, **c** Response of optimal photochemical efficiency of PSII (Fv/Fm). **d** Changes of non-photochemical quenching (NPQ), photochemistry quenching (qP) and effective quantum yields of PSII (ΦPSII). **e**The change of photosynthetic electron transport (ETR)

Grape plantlets were cultured for 25 days at 1000 μmol·mol^− 1^ of CO_2_ and compare with control conditions. The results showed that the leaf area, plantlet height and shoot fresh weight increased significantly in Cs and C0 compared with CK (Additional file 1: Table S1). In addition, the number of adventitious roots in tubers also was increased in Cs and C0 (Fig. 1a).

In compared to CK, the Fv/Fm decreased in Cs and C0, and significantly lower in C0 than that of CK (Fig. 1b, c). The qP and ETR rised in Cs and C0 (Fig. 1d, e). The ETR of C0 was significantly higher than the Cs and CK. The qP of C0 was significantly higher than Cs and CK (Fig. 1d). The NPQ was in the following order: Cs < C0 < CK (Fig. 1d). The decrease of NPQ indicated that eCO_2_ enhanced the efficiency of PSII and reduced the damage caused by biotic and abiotic stress. The ΦPSII of Cs and C0 were significantly higher than CK (Fig. 1d). These results suggested that eCO_2_ improved photosynthesis and reflected by chlorophyll fluorescence parameters, including Fv/Fm, ETR, qP, NPQ and ΦPSII.

### Transcriptome and proteome differences expression in eCO_2_

**Fig. 2:**
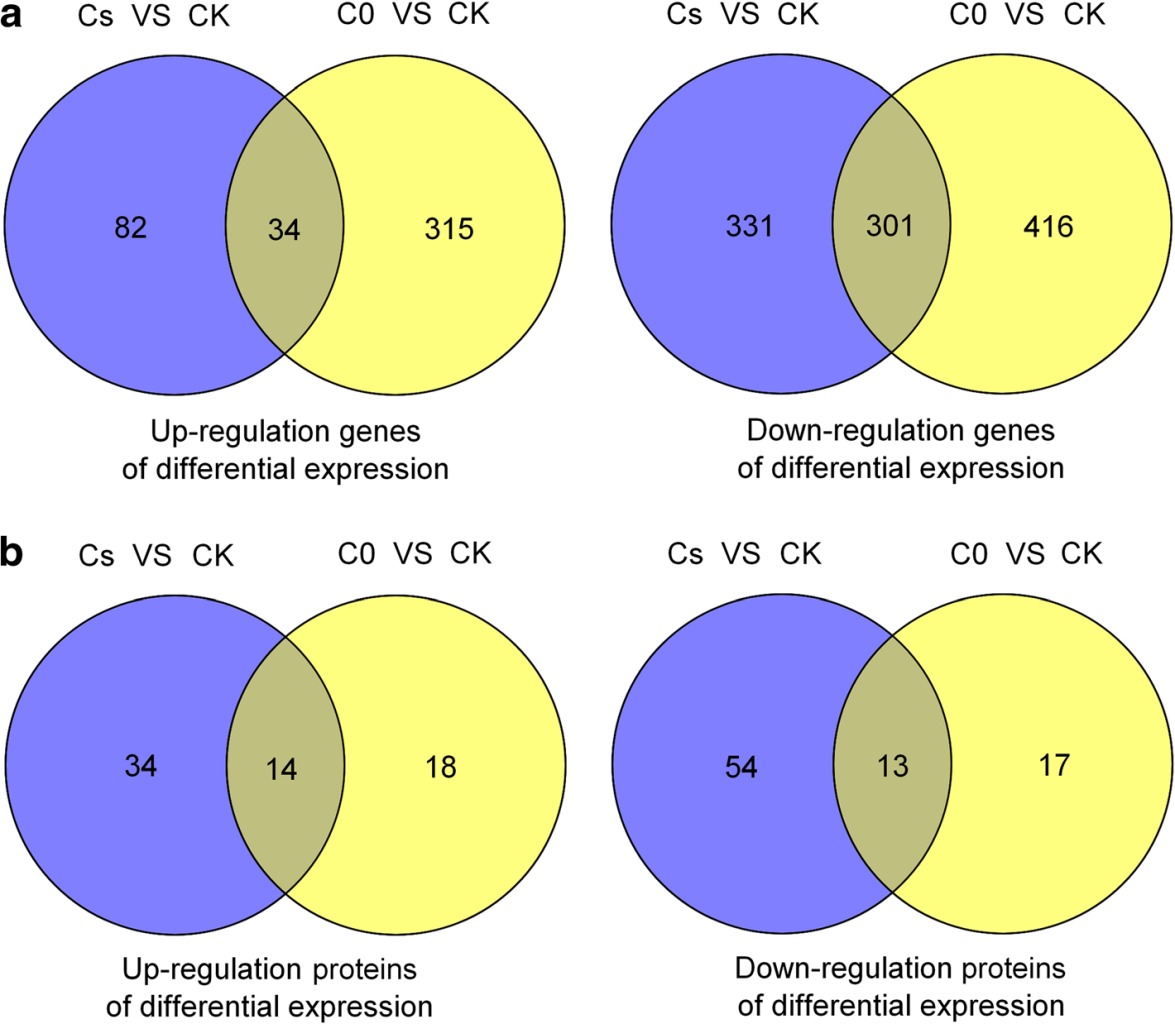
Venn diagram showing of number of DEGs and DEPs expressed in different treatments. **a** The DEGs identified with Cs versus CK and C0 versus CK. **b** The DEPs identified with Cs versus CK and C0 versus CK

In the transcriptome project, three RNA-Seq groups with three replications were sequenced, 29.5Gb clean bases were generated from the 9 libraries. After data processing, 46.49–47.46 million high-quality reads were obtained (Table 1). Through transcriptome analysis, a total of 1814 DEGs were observed by comparing with CK, of which 116 up-regulated and 632 down-regulated DEGs were identified in Cs versus CK, 349 up-regulated and 717 down-regulated DEGs were identified in C0 versus CK (Fig. 2a). According to SDS-PAGE analysis, protein sample could be tested in the next step (Additional file 2: Figure S1). After analysis of proteomic profiling, a total of 177 DEPs were observed from the pooled data for above two comparison groups. Among them, 48 up-regulated DEPs and 67 down-regulated DEPs were identified in Cs versus CK, 32 up-regulated DEPs and 30 down-regulated DEPs were identified in C0 versus CK (Fig. 2b).

**Table 1:** Summary of transcriptome sequencing data of *Vitis vinifera* L. cv. ‘Pinot Noir’ leaves transcriptome

	CK	C0	Cs
Total reads	46,888,137	47,455,710	46,499,479
Total Mapped	42,923,887 (91.55%)	43,235,905 (91.15%)	42,843,192 (92.14%)
Multiple mapped	1,017,694 (2.17%)	1,009,825 (2.12%)	982,852 (2.11%)
Uniquely mapped	41,906,193 (89.38%)	42,226,081 (89.02%)	41,860,340 (90.02%)
Read-1	20,981,121 (44.75%)	21,144,642 (44.59%)	20,980,375 (45.12%)
Read-2	20,925,072 (44.63%)	21,081,439 (44.43%)	20,879,965 (44.90%)
Reads map to ‘+’	20,973,998 (44.73%)	21,131,755 (44.55%)	20,957,526 (45.07%)
Reads map to ‘-’	20,932,195 (44.64%)	21,094,326 (44.47%)	20,902,814 (44.95%)
Non-splice reads	25,189,915 (53.72%)	25,355,802 (53.26%)	25,331,236 (54.48%)
Splice reads	16,716,278 (35.65%)	16,870,279 (35.76%)	16,529,104 (35.54%)
Reads mapped in proper pairs	40,350,237 (86.06%)	40,714,512 (85.88%)	40,408,286 (86.90%)

### GO analysis of DEGs and DEPs

Of the 25,679 genes identified in the transcriptome analysis, 17,750 genes (69.12%) were annotated via GO analysis. Compared with CK, 748 DEGs identified in Cs were enriched in the biological process (BP), cellular component (CC), and molecular function (MF) categories. In the cellular components category, most of DEGs were involved in integral component of membrane (99 genes) and cytoplasm (94 genes). In the biological process category, most of DEGs were involved in defense response (70 genes) and transcription, DNA-templated (59 genes). In the molecular function category, most of DEGs were involved in transcription factor activity, sequence-specific DNA binding (54 genes) and ATP binding (48 genes) (Additional file 3: Table S2 A).

The 1066 DEGs of C0 versus CK were detected. Most DEGs mainly enriched in cytoplasm (98 genes) and integral component (97 genes) of cellular components. In the biological process category, most of DEGs were involved in defense response (53 genes) and transcription, DNA-templated (48 genes). In the molecular function category, most of DEGs were involved in metal ion binding (52 genes) and transcription factor activity, sequence-specific DNA binding (45 genes) (Additional file 3: Table S2 B).

From the pooled data for Cs versus CK, 115 DEPs were enriched in cell part (68 proteins) of cellular components. In the biological process category, most of DEPs were involved in metabolic process (81 proteins). In the molecular function category, most of DEPs were involved in catalytic activity (59 proteins) (Additional file 4: Table S3 A). Among the top 10 up-regulated DEPs, there were 3 proteins with abundance change related to photosynthesis: oxygen-evolving enhancer protein 3 (PsbQ; XP_002275624.1), chlorophyll a-b binding protein CP26 (Lhcb5; XP_002264295.1), photosystem II protein V (PsbE; YP_567093.1) (Table 2). But ribulose bisphosphate carboxylase/oxygenase activas (Rca; XP_002282979.1) was the 11th top up-regulated DEPs. Among the top 10 down-regulated DEPs, 2 proteins related to secondary metabolites: aspartokinase 2 (XP_010660689.1) and protein luteindeficient 5 (XP_002279984.3); one protein related to polysaccharide catabolic process: inactive beta-amylase 9 (XP_002276777.1); one protein related to stress tolerance: acid phosphatase 1 (XP_003632911.1) (Table 2).

**Table 2:** Summary of the top 10 DEPs at Cs

Accession	Description	Fold Change (Cs/CK)	KEGG term	*P* value
XP_002274242.1	PREDICTED: major allergen Pru av. 1 [*Vitis vinifera*]	2.25	none	1.52E-07
XP_002276622.1	PREDICTED: calvin cycle protein CP12–3, chloroplastic [Vitis vinifera]	2.05	none	2.226E-06
XP_002266659.1	PREDICTED: DNA ligase 1 isoform X1 [Vitis vinifera]	1.87	none	0.0062579
XP_002285919.1	PREDICTED: dehydrin ERD14 [Vitis vinifera]	1.82	none	2.481E-05
XP_002273316.1	PREDICTED: protein EXORDIUM-like 2 [Vitis vinifera]	1.76	none	1.736E-06
XP_002275624.1	PREDICTED: oxygen-evolving enhancer protein 3, chloroplastic [Vitis vinifera]	1.76	Photosynthesis (ko00195)	2.678E-08
XP_003631204.1	PREDICTED: MLP-like protein 34 [Vitis vinifera]	1.73	none	2.737E-05
NP_001267958.1	aquaporin TIP2;1 [Vitis vinifera]	1.69	none	4.675E-06
XP_002264295.1	PREDICTED: chlorophyll a-b binding protein CP26, chloroplastic [Vitis vinifera]	1.68	Photosynthesis - antenna proteinsko (00196)	3.238E-09
YP_567093.1	photosystem II protein V (chloroplast) [Vitis vinifera]	1.67	Photosynthesis (ko00195)	4.256E-07
XP_003631660.1	PREDICTED: protein RADIALIS-like 1 [Vitis vinifera]	0.36	none	0.0002393
XP_010660689.1	PREDICTED: aspartokinase 2, chloroplastic isoform X1 [Vitis vinifera]	0.44	Glycine, serine and threonine metabolism/Cysteine and methionine metabolism/Lysine biosynthesis/Monobactam biosynthesis (ko00260;ko00270;ko00300;ko00261)	5.242E-06
XP_002276777.1	PREDICTED: inactive beta-amylase 9 [Vitis vinifera]	0.456	none	3.727E-05
XP_003632911.1	PREDICTED: acid phosphatase 1 [Vitis vinifera]	0.47	none	3.221E-08
XP_002277279.1	PREDICTED: GDSL esterase/lipase At5g45950 [Vitis vinifera]	0.48	none	4.556E-06
XP_002284222.1	PREDICTED: ribonuclease III domain-containing protein RNC1, chloroplastic [Vitis vinifera]	0.50	none	9.809E-06
XP_002279984.3	PREDICTED: protein LUTEIN DEFICIENT 5, chloroplastic [Vitis vinifera]	0.51	Carotenoid biosynthesis (ko00906)	0.0001186
XP_003634206.1	PREDICTED: thaumatin-like protein [Vitis vinifera]	0.52	none	3.04E-05
XP_002281642.1	PREDICTED: 29 kDa ribonucleoprotein A, chloroplastic [Vitis vinifera]	0.52	none	3.048E-06
XP_002265252.1	PREDICTED: tetrapyrrole-binding protein, chloroplastic [Vitis vinifera]	0.53	none	1.121E-06

A total of 62 DEPs were detected in C0 versus CK. DEPs were annotated and enriched in the three categories. In the cellular components, most proteins were involved in cell part (36 proteins). In the biological process category, most proteins were involved in metabolic process (45 proteins). In the molecular function category, most proteins were involved in catalytic activity (28 proteins) (Additional file 4: Table S3 B). Among the top 10 up-regulated DEPs, 5 of the top 10 DEPs were photosynthesis proteins: oxygen-evolving enhancer protein 3 (PsbQ; XP_002275624.1), Plastocyanin (Pc; XP_002285904.1), Chlorophyll a-b binding protein (Lhcb6; XP_002263201.1), chlorophyll a-b binding protein CP26 (Lhcb5; XP_002264295.1) and chlorophyll a-b binding protein of LHCII type 1 (Lhcb1; XP_002283566.1) (Table 3). The Rca (XP_002282979.1) was the 13th top up-regulated DEPs. Three of the top 10 DEPs down-regulated proteins: bifunctional 3-dehydroquinate dehydratase/shikimate dehydrogenase (XP_002270232.1, XP_002270188.1) and beta-glucosidase 13 (XP_002270422.2) (Table 3) were associated with biosynthesis of secondary metabolites.

**Table 3:** Summary of the top 10 DEPs at C0

Accession	Description	Fold Change(C0/CK)	KEGG term	*P* value
XP_002274242.1	PREDICTED: major allergen Pru av. 1 [Vitis vinifera]	2.00	none	4.049E-07
XP_002275624.1	PREDICTED: oxygen-evolving enhancer protein 3, chloroplastic [Vitis vinifera]	1.91	Photosynthesis (ko00195)	2.24E-08
XP_002285904.1	PREDICTED: plastocyanin [Vitis vinifera]	1.88	Photosynthesis (ko00195)	6.998E-08
XP_002263201.1	PREDICTED: chlorophyll a-b binding protein CP24 10A, chloroplastic [Vitis vinifera]	1.75	Photosynthesis - antenna proteins (ko00196)	3.512E-07
XP_002279607.1	PREDICTED: sec-independent protein translocase protein TATA, chloroplastic [Vitis vinifera]	1.70	Bacterial secretion system/Protein export (ko03070; ko03060)	0.0005
XP_002267428.1	PREDICTED: patellin-3 [Vitis vinifera]	1.69	none	5.44E-06
XP_010653784.1	PREDICTED: uncharacterized protein LOC100245204 isoform X1 [Vitis vinifera]	1.66	none	0.0001
XP_019076764.1	PREDICTED: metal transporter Nramp3 isoform X2 [Vitis vinifera]	1.66	Ferroptosis/Lysosome (ko04216;ko04142)	2.638E-06
XP_002263064.1	PREDICTED: plasma membrane-associated cation-binding protein 1 [Vitis vinifera]	1.61	none	2.445E-06
XP_002264295.1	PREDICTED: chlorophyll a-b binding protein CP26, chloroplastic [Vitis vinifera]	1.59	Photosynthesis-antenna proteins (ko00196)	1.856E-09
XP_002277053.2	PREDICTED: GDSL esterase/lipase At1g09390 [Vitis vinifera]	0.55	none	3.675E-05
XP_019073045.1	PREDICTED: 50S ribosomal protein L24, chloroplastic [Vitis vinifera]	0.59	Ribosome (ko03010)	6.23E-07
XP_002283566.1	PREDICTED: chlorophyll a-b binding protein of LHCII type 1 [Vitis vinifera]	0.62	Photosynthesis - antenna proteins (ko00196)	0.0009
XP_002274256.1	PREDICTED: limonoid UDP-glucosyltransferase [Vitis vinifera]	0.63	none	0.00012
XP_002270232.1	PREDICTED: bifunctional 3-dehydroquinate dehydratase/shikimate dehydrogenase, chloroplastic [Vitis vinifera]	0.63	Phenylalanine, tyrosine and tryptophan biosynthesis (ko00400)	6.293E-05
XP_002270188.1	PREDICTED: bifunctional 3-dehydroquinate dehydratase/shikimate dehydrogenase, chloroplastic [Vitis vinifera]	0.65	Phenylalanine, tyrosine and tryptophan biosynthesis (ko00400)	3.948E-07
XP_003634206.1	PREDICTED: thaumatin-like protein [Vitis vinifera]	0.65	none	8.466E-05
NP_001268023.1	lipoxygenase [Vitis vinifera]	0.65	Linoleic acid metabolism/alpha-Linolenic acid metabolism ko00591; ko00592	4.482E-07
XP_002285526.1	PREDICTED: transmembrane 9 superfamily member 9 [Vitis vinifera]	0.65	none	1.907E-05
XP_002270422.2	PREDICTED: beta-glucosidase 13 [Vitis vinifera]	0.65	Phenylpropanoid biosynthesis/Starch and sucrose metabolism /Cyanoamino acid metabolism (ko00940; ko00500; ko00460)	0.0001

### KEGG pathway analysis for DEPs

To further investigate the plant reaction to eCO_2_, DEPs were identified by searching the KEGG database. The 115 DEPs of Cs were assigned to 52 KEGG pathways, and the top 5 pathways with the highest rich factor were photosynthesis-antenna proteins, metabolic pathways, biosynthesis of secondary metabolites, carbon metabolism, biosynthesis of amino acids (Additional file 5: Table S4 A). The 62 DEPs of C0 were assigned to 20 KEGG pathways, and the top 5 pathways with the highest rich factor were photosynthesis-antenna proteins, photosynthesis, metabolic pathways, phenylpropanoid biosynthesis, phenylalanine, tyrosine and tryptophan biosynthesis (Additional file 5: Table S4 B).

The common pathways with the highest rich factor of Cs versus CK and C0 versus CK were photosynthesis-antenna proteins, photosynthesis and metabolic pathways. Simultaneously, 9, 6 and 48 proteins were involved in the three pathways, respectively. Moreover, 12 proteins involved in metabolic pathway were overlaps with photosynthesis (Table 4).

**Table 4:** DEPs affected by eCO_2_ in KEGG pathway analysis in *Vitis vinifera* L. cv. ‘Pinot Noir’ leaves

UniProt ID	Accession	Description	Fold Change		*P* value	
			Cs/CK	C0/ CK	Cs/ CK	C0/ CK
Photosynthesis - antenna proteins						
A5ASW8	XP_002263201.1	chlorophyll a-b binding protein CP24 10A, chloroplastic [Vitis vinifera]	1.61	1.75	2.56E-07	3.51E-07
A5BAI4	XP_003633024.1﻿	Chlorophyll a-b binding protein, chloroplastic [Vitis vinifera]	0.94	1.39	0.0321	0.0001
A5ASG6	XP_002271687.1	chlorophyll a-b binding protein 151, chloroplastic [Vitis vinifera]	1.46	0.94	9.56E-06	0.0177
F6I5I9	XP_002273201.1	photosystem I chlorophyll a/b-binding protein 3–1, chloroplastic [Vitis vinifera]	1.50	1.50	5.03E-08	4.30E-08
A5BW14	XP_002274150.2	chlorophyll a-b binding protein 13, chloroplastic [Vitis vinifera]	1.49	1.49	1.40E-07	1.07E-07
F6HMH7	XP_002275075.1	chlorophyll a-b binding protein of LHCII type 1 [Vitis vinifera]	1.45	1.5	6.43E-08	3.28E-07
F6H2E4	XP_002284493.1	chlorophyll a-b binding protein 13, chloroplastic [Vitis vinifera]	1.42	1.46	2.60E-06	5.47E-06
A5C4U9	XP_002285646.1	chlorophyll a-b binding protein of LHCII type 1 [Vitis vinifera]	1.62	0.66	0.0006	0.0003
A5BPB2	XP_002283566.1	chlorophyll a-b binding protein of LHCII type 1 [Vitis vinifera]	1.36	0.62	0.0014	0.0009
Photosynthesis						
F6H8B4	XP_002275624.1	oxygen-evolving enhancer protein 3, chloroplastic [Vitis vinifera]	1.76	1.91	2.68E-08	2.24E-08
E0CQV6	XP_002285904.1	Plastocyanin [Vitis vinifera]	0.59	1.88	6.97E-06	6.99E-08
Q0ZJ25	YP_567071.1	photosystem II protein D2, chloroplastic [Vitis vinifera]	1.48	1.24	6.80E-09	1.27E-06
Q0ZJ03	YP_567093.1	photosystem II protein V, chloroplastic [Vitis vinifera]	1.67	1.57	4.26E-07	7.82E-07
F6HVW3	XP_002274963.1	ATP synthase delta chain, chloroplastic [Vitis vinifera]	1.30	1.42	5.11E-09	3.35E-09
F6I0D9	XP_003631913.1	photosystem I reaction center subunit N, chloroplastic [Vitis vinifera]	1.37	1.42	4.83E-08	3.98E-09
Metabolic pathway						
D0VBC1	NP_001268000.1﻿	3-deoxy-D-arabino-heptulosonate-7-phosphate synthase 03 [Vitis vinifera]	0.73	0.85	1.32E-05	0.0001
A5ASW8	XP_002263201.1﻿	Chlorophyll a-b binding protein, chloroplastic [Vitis vinifera]	1.61	1.71	2.56E-07	3.51E-07
O22519	NP_001268064.1	chalcone synthase [Vitis vinifera]	1.16	0.66	0.0027	3.91E-05
A5BAI4	XP_003633024.1﻿	Chlorophyll a-b binding protein, chloroplastic [Vitis vinifera]	0.94	1.39	0.0321	0.0001
D7TIY1	XP_002264311.2﻿	Threonine dehydratase [Vitis vinifera]	0.67	0.79	4.02E-05	0.0004
F6HTH9	XP_002267374.1	bifunctional riboflavin biosynthesis protein RIBA 1, chloroplastic [Vitis vinifera]	0.68	0.87	4.02E-06	0.0002
F6H7K5	XP_002267414.1	thiamine thiazole synthase 2, chloroplastic [Vitis vinifera]	0.69	0.81	8.41E-08	8.09E-07
A5B8T3	XP_002268097.1﻿	fructokinase-2-like [Vitis vinifera]	0.62	0.85	8.58E-07	1.59E-05
D7U461	XP_002279832.1﻿	probable mannitol dehydrogenase [Vitis vinifera]	0.78	0.90	0.0095	0.1358
D7TUX2	XP_002270188.1﻿	bifunctional 3-dehydroquinate dehydratase/shikimate dehydrogenase, chloroplastic [Vitis vinifera]	0.55	0.65	2.21E-08	3.95E-07
D7UBU8	XP_002270736.1	cytochrome P450 77A2 [Vitis vinifera]	0.61	0.80	2.45E-05	0.0004
D7T9N2	XP_002276048.1	UDP-N-acetylglucosamine diphosphorylase 1 [Vitis vinifera]	0.94	0.90	0.0238	0.0012
A5ASG6	XP_002271687.1﻿	chlorophyll a-b binding protein 151, chloroplastic-like [Vitis vinifera]	1.46	0.94	9.56E-06	0.0177
F6HIF0	NP_001267871.1	aconitase 2, mitochondria [Vitis vinifera]	1.00	1.07	0.6560	0.0089
A5BW14	XP_002274150.2﻿	chlorophyll a-b binding protein 13, chloroplastic [Vitis vinifera]	1.49	1.49	1.40E-07	1.07E-07
F6HMH7	XP_002275075.1	chlorophyll a-b binding protein of LHCII type 1 [Vitis vinifera]	1.45	1.5	6.43E-08	3.28E-07
F6I639	XP_002275348.1﻿	probable glycerol-3-phosphate acyltransferase 8 [Vitis vinifera]	0.70	0.91	3.15E-06	0.0025
F6H8B4	XP_002275624.1	oxygen-evolving enhancer protein 3, chloroplastic [Vitis vinifera]	1.76	1.91	2.68E-08	2.24E-08
F6HA36	XP_002275678.1﻿	L-ascorbate oxidase [Vitis vinifera]	0.64	1.17	0.0015	0.0344
F6HG44	XP_002270414.1	glyceraldehyde-3-phosphate dehydrogenase B, chloroplastic [Vitis vinifera]	1.11	0.99	0.0112	0.7452
F6HDH8	XP_002276777.1	beta-amylase 1, chloroplastic [Vitis vinifera]	0.46	0.95	3.73E-05	0.0009
A5C718	XP_002276967.1﻿	ribulose bisphosphate carboxylase small chain, chloroplastic [Vitis vinifera]	1.40	1.31	8.83E-09	5.77E-08
F6HKX6	XP_002277825.3﻿	acetyl-coenzyme A carboxylase carboxyl transferase subunit alpha, chloroplastic [Vitis vinifera]	0.70	0.81	3.21E-07	5.36E-06
F6HES4	XP_002278339.1﻿	GDP-L-galactose phosphorylase 2 [Vitis vinifera]	0.65	0.70	4.62E-07	2.80E-07
F6HDW1	XP_002279975.1﻿	pyruvate kinase isozyme A, chloroplastic [Vitis vinifera]	0.66	0.81	2.26E-06	7.81E-05
A5AGN5	XP_002280094.1﻿	ketol-acid reductoisomerase, chloroplastic-like [Vitis vinifera]	0.68	0.87	3.24E-08	1.19E-06
F6I397	XP_002280760.1	transketolase, chloroplastic [Vitis vinifera]	0.71	0.81	1.22E-06	9.10E-06
F6H521	XP_002281731.1﻿	peroxidase P7 [Vitis vinifera]	1.48	1.33	0.0002	0.0007
F6H042	XP_002283364.1﻿	geranylgeranyl pyrophosphate synthase, chloroplastic [Vitis vinifera]	0.66	0.92	0.0002	0.02701
F6H2E4	XP_002284493.1﻿	chlorophyll a-b binding protein 13, chloroplastic [Vitis vinifera]	1.42	1.46	2.59E-06	5.47E-06
E0CSP0	XP_002284769.2	protochlorophyllide reductase, chloroplastic [Vitis vinifera]	0.70	0.86	0.0008	0.0084
A5BZY3	XP_002285583.1	glutamyl-tRNA reductase 1, chloroplastic-like [Vitis vinifera]	0.62	0.81	2.28E-06	3.96E-05
A5C4U9	XP_002285646.1﻿	chlorophyll a-b binding protein of LHCII type 1 [Vitis vinifera]	1.62	0.66	0.0006	0.0003
D7SYQ0	XP_010646454.1	acetolactate synthase small subunit 2, chloroplastic [Vitis vinifera]	0.69	0.78	4.67E-05	0.0003
F6HA09	XP_010651495.1	serine--glyoxylate aminotransferase [Vitis vinifera]	1.47	1.28	6.07E-09	5.81E-08
D7SVZ9	XP_010652823.1	inositol-3-phosphate synthase [Vitis vinifera]	1.42	1.26	5.87E-09	4.29E-08
A5C6H7	XP_002271896.1	sucrose synthase 2 [Vitis vinifera]	1.01	0.99	0.8398	0.6544
F6HWQ2	XP_010656841.1﻿	aspartokinase 1, chloroplastic [Vitis vinifera]	0.86	0.86	0.0002	0.0002
E0CUM8	XP_010662621.1﻿	plastidial pyruvate kinase 2 [Vitis vinifera]	0.68	0.80	2.87E-05	0.0002
A5CAL1	XP_003632860.1	hydroxyphenylpyruvate reductase [Vitis vinifera]	0.99	0.87	0.4973	5.87E-06
D7UCD0	XP_019081328.1	bifunctional L-3-cyanoalanine synthase/cysteine synthase 1, mitochondrial [Vitis vinifera]	1.45	1.23	1.46E-08	4.39E-06
Q0ZJ25	YP_567071.1	photosystem II protein D2 [Vitis vinifera]	1.48	1.24	6.80E-09	1.27E-06
Q0ZJ03	YP_567093.1	photosystem II protein V,chloroplast [Vitis vinifera]	1.67	1.57	4.26E-07	7.82E-07
D7T2U5	XP_002270422.2	beta-glucosidase 13 [Vitis vinifera]	0.87	0.65	0.0060	0.0001
F6HVW3	XP_002274963.1﻿	ATP synthase delta chain, chloroplastic [Vitis vinifera]	1.30	1.42	5.11E-09	3.35E-09
A5BPT8	XP_002285277.1	phenylalanine ammonia-lyase-like [Vitis vinifera]	0.93	0.71	0.0104	0.0002
F6I0D9	XP_003631913.1﻿	photosystem I reaction center subunit N, chloroplastic [Vitis vinifera]	1.37	1.42	4.83E-08	3.98E-09
F6H0Z0	XP_003634480.1﻿	cationic peroxidase 1 [Vitis vinifera]	1.11	1.51	0.0001	6.57E-06

**Fig. 3:**
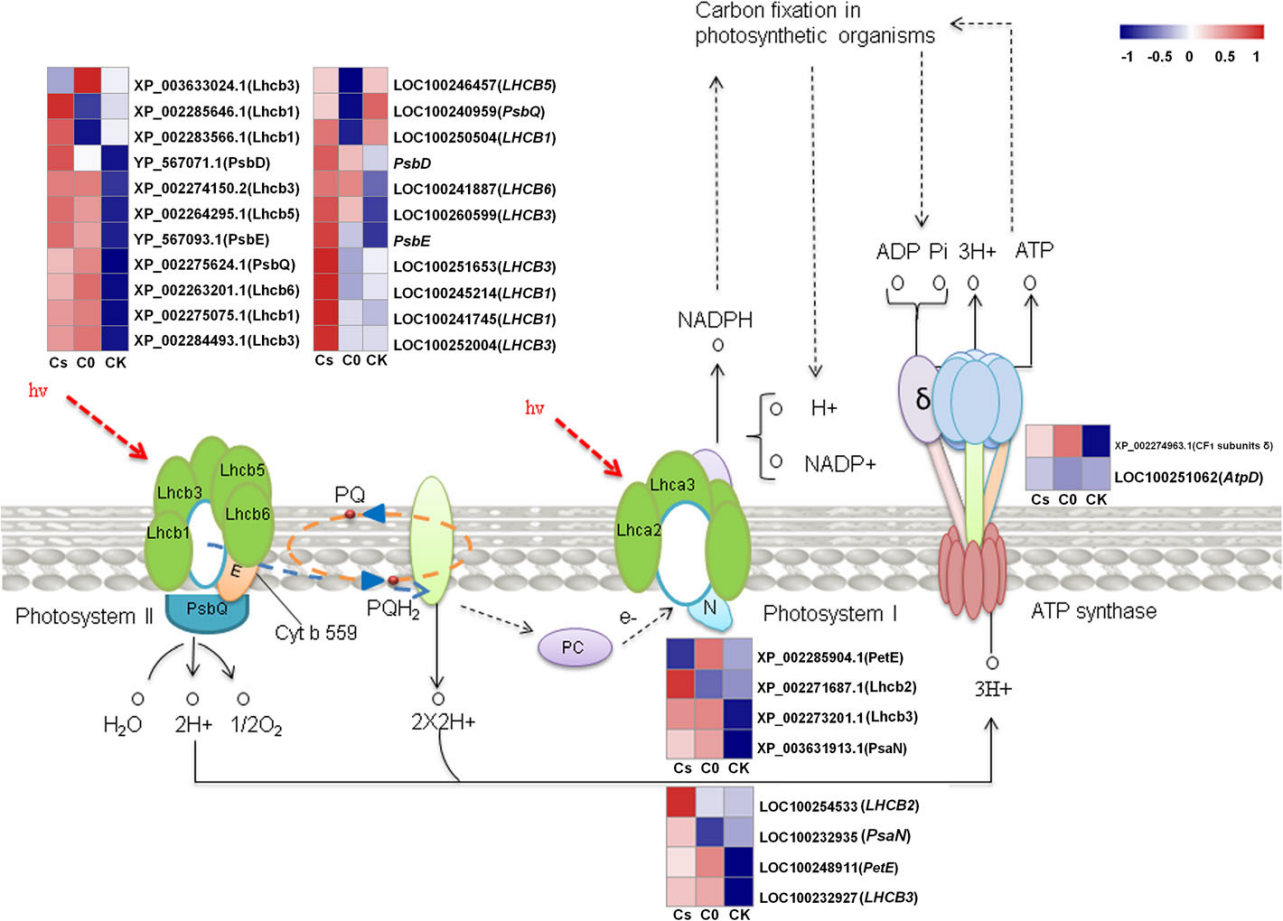
The DEPs and their corresponding genes expression of photosynthesis system

**Fig. 4:**
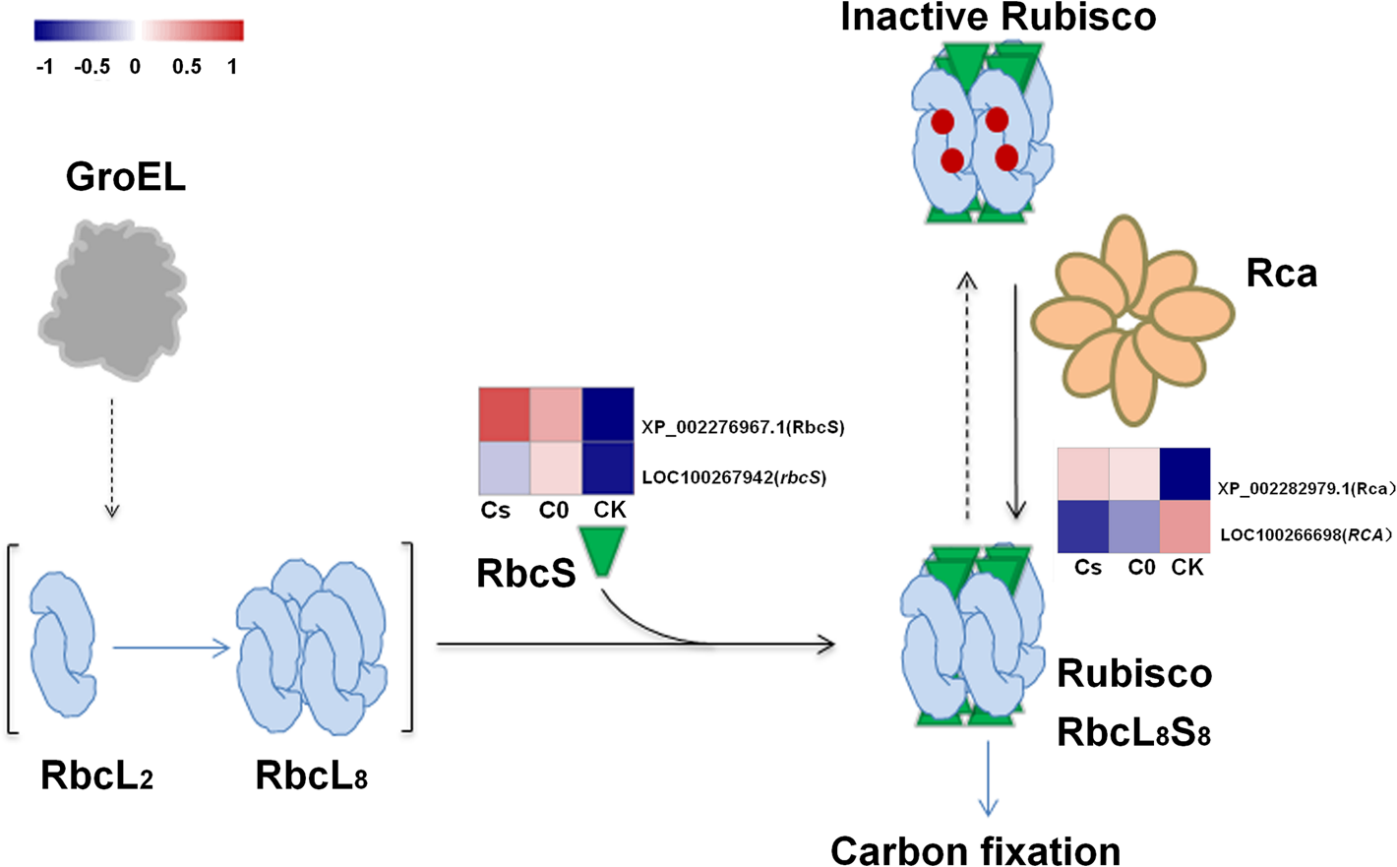
The expression of DEPs and their corresponding genes in formation of Rubisco

There were 8 chlorophyll a-b binding proteins: Chlorophyll a-b binding protein (Lhcb6, XP_002263201.1), chlorophyll a-b binding protein 151 (Lhcb2; XP_002271687.1), photosystem I chlorophyll a-b-binding protein 3–1 (Lhcb3; XP_002273201.1), chlorophyll a-b binding protein 13 (Lhcb3; XP_002274150.2), chlorophyll a-b binding protein of LHCII type 1 (Lhcb1; XP_002275075.1), chlorophyll a-b binding protein 13 (Lhcb3; XP_002284493.1), chlorophyll a-b binding protein of LHCII type 1 (Lhcb1; XP_002285646.1) and chlorophyll a-b binding protein of LHCII type 1 (Lhcb1; XP_002283566.1) significantly up-regulated in Cs and C0 compared with those in CK, only 1 protein, Chlorophyll a-b binding protein (Lhcb3; XP_003633024.1) was descend in Cs (Fig. 3). There were 4 subunits of PSII: PsbQ (XP_002275624.1), PsbE (YP_567093.1), photosystem II protein D2 (PsbD; YP_567071.1) and photosystem I reaction center subunit N (PsaN; XP_003631913.1) significantly up-regulated in Cs and C0, but 1 subunit Pc (XP_002285904.1) of PSI was down-regulated (Fig. 3). Ribulose bisphosphate carboxylase small chain (RbcS; XP_002276967.1) and ATP synthase delta chain (XP_002274963.1) were up-regulated in Cs and C0 (Figs. 3, 4). Other proteins: beta-glucosidase 13 (XP_002270422.2), beta-amylase 1 (XP_002276777.1), threonine dehydratase (XP_002264311.2) and GDP-L-galactose phosphorylase 2 (XP_002278339.1) involved in metabolic were down-regulated in Cs and C0 compared with those in CK, and also involved in polysaccharide catabolic process and biosynthesis of secondary metabolites (Table 4).

### Combined analysis of transcriptome and proteome data

To reveal eCO_2_ regulates photosynthesis gene via transcript and protein levels, the transcript data were used to analyze 18 DEPs associated with photosynthesis and metabolic pathways. Seven DEPs: Lhcb6 (XP_002263201.1), Lhcb3 (XP_002273201.1, XP_002284493.1), Lhcb1 (XP_002275075.1, XP_002285646.1, XP_002283566.1) and RbcS (XP_002276967.1) and their mRNA expression showed up-regulated in Cs and C0. However, there were 3 DEPs, PsbQ (XP_002275624.1), Rca (XP_002282979.1) and Lhcb5 (XP_002264295.1) involved in photosynthesis up-regulated in Cs and C0, but their mRNA down-regulated. Other 8 DEPs were not correlated with genes expression under eCO_2_ (Figs. 3, 4).

The RbcS (XP_002276967.1) and its corresponding gene were up-regulated in Cs and C0. ATP synthase delta chain (XP_002274963.1) and Rca (XP_002282979.1) were up-regulated but their corresponding genes were down-regulated in Cs and C0 (Figs. 3, 4). The results make clear that eCO_2_ have different effects on gene transcription and translation. RbcS was not correlated with its mRNA level, suggesting that the change in the amount of RbcS is regulated at their transcript levels by eCO_2_. However, Rca was negatively correlated with its mRNA level, it is suggested that the change in the amount of its corresponding protein is regulated at their translation levels by eCO_2_.

### Confirmation of qRT-PCR

**Fig. 5:**
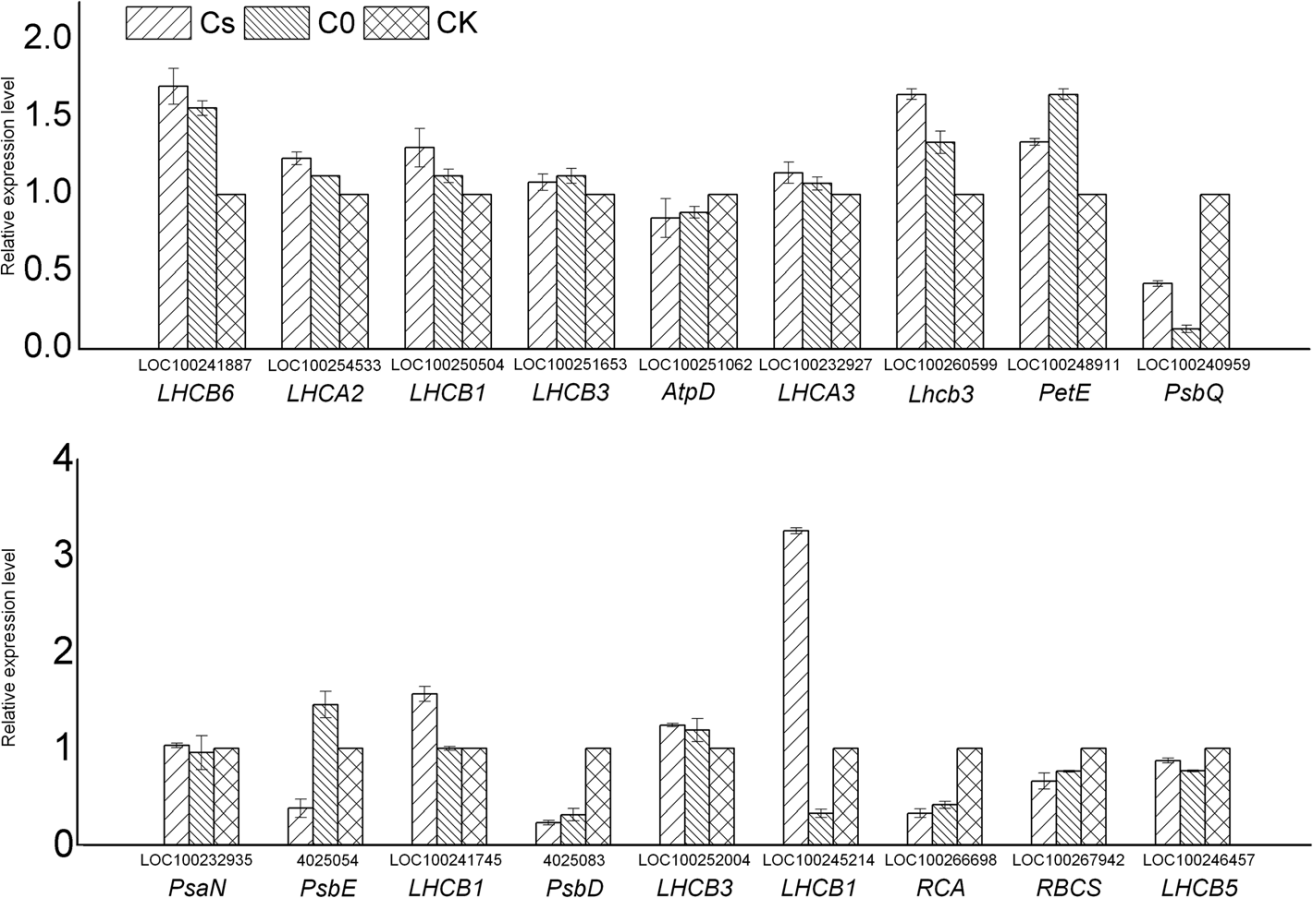
qRT-PCR validation of the relative expression levels of 18 slected genes from Cs, C0 and CK in leaves

In order to evaluate our transcriptome-sequencing data, 18 genes in the photosynthesis and metabolic pathway were selected for qRT-PCR. The results analyse indicated that 15 genes (83.33%) showed similar trends in the relative expression levels, which suggested that the gene expression changes detected by transcriptome-sequencing analysis were reliable. But 3 genes (16.67%) analyzed by qRT-PCR, i.e., *PsbE* (4025054), *PsbD* (4025083) and *LHCB3* (LOC100252004) were not consistent with our RNA-seq data (Fig. 5).

## Discussion

### Proteins involved in photosynthesis were regulated by eCO_2_

Light-harvesting complexes (LHC) of photosynthetic plant bind pigments essential for augmenting light capture and photoprotection [31]. LHCI and LHCII belong to photosystem I (PSI) and photosystem II (PSII), respectively. LHCII is a trimeric light-harvesting complex (Lhc) composed of a combination of the *Lhcb* gene products and others [32]. Plants can develop strategies of acclimating varies light conditions during seasons and can rapidly adjust photosynthesis antenna sizes in case of excess light, avoiding over excitation and formation of harmful by products [33]. CO_2_ concentration could affect the primary light reaction of photosynthesis in soybean leaves [34]. In our research, 8 proteins of LHCII were up-regulated in eCO_2_ (Table 4), this change indicated that eCO_2_ could induce more light-harvesting proteins (Fig. 3), and cause an increase in the size of the PSI and PSII antenna. The light-harvesting complex II (LHCII) could convert most photons to biochemical energy and biomass [35]. With the increase of LHCII in eCO_2_, more light energy can be absorbed and converted into photosystem. The increase of qP and decrease of NPQ confirming that leaves could absorb more light energy under eCO_2_ (Fig. 1d). In present study, the number of up-regulated light-harvesting proteins of PSII was more than that of PSI, which showed that the eCO_2_ had a great influence on PSII. Additionally, the LHCII conditions would migration from PSII to PSI under deficient CO_2_ environment [36]. CP24 was up-regulated in eCO_2_, it was essential for connecting LHCII to the PSII complex [37, 38]. Lack of the light-harvesting complex CP24 affects the structure and function of the grana membranes of higher plant chloroplasts [37]. Overall, these proteins, which were up-regulated under eCO_2_, could absorb and convert more light energy into the photosystem.

In photosynthesis pathway, the expression of PetE (XP_002285904.1) and Chlorophyll a-b binding protein (XP_003633024.1) were descend in Cs. Interestingly, the expression of *PetE* (LOC100248911) and *LHCB3* (LOC100252004) were ascend in Cs and C0. These results indicated that most of the DEPs and their corresponding genes expression were inconsistent. The eCO_2_ may cause various modifications of related proteins after translation and needs to be study for further.

### eCO_2_ regulates metabolic protein expression

There were 48 DEPs involved in metabolic pathway, while 12 of them were overlaps with photosynthesis. This might indicate that eCO_2_ would affect other metabolic through adjusting photosynthesis. Our results indicated many of down-regulated DEPs were enriched in metabolic pathway in eCO_2_, which were related to biosynthesis of secondary metabolites (Table 4). This change is suggesting that eCO_2_ probably decreased biosynthesis of secondary metabolites [39]. Therefore, plant could accumulation more primary metabolism products to encourage growth.

The eCO_2_ could ameliorate the effects caused by drought [40], high temperatures [41], and maintaining higher photosynthetic rates. This may be linked to the reduction in stomatal conductance [42]. Moreover, increasing photosystem antenna size must inevitably cause structural changes needed to ensure high efficiency of its functioning [43]. There were 4 DEPs (PsbQ, PsbE, PsbD and PsaN; Fig. 3) up-regulated in eCO_2_. Those proteins could maintain the stability of the photosystem reaction center [44, 45]. By analyzing changes of those proteins in eCO_2_, we can conclude that eCO_2_ could trigger some proteins to maintain the stability of the photosynthesis system. Therefore, eCO_2_ could ameliorate the adverse effect under abiotic stress. PsbQ can increase PSII activity and stability of oxygen release complexes (OECs) [45]. It is also the water decomposition subunit [46]. The PsbQ was up-regulated in eCO_2_, this means eCO_2_ could promote water decomposition and maintain stability in OECs by regulating PsbQ. The other 3 proteins (PsbE, PsbD and PsaN) related to photosynthetic electron transport and accumulation of photosynthetic substances [44]. Those proteins (LHCs, PsbQ, PetE, PsbD, PsaN) increased in eCO_2_ (Fig. 3), resulting in absorbing more light energy and promoting more photosynthetic electron transport. This is causing the advance of qP and ETR, and the reduction of NPQ (Fig. 1d, e).

ATP synthase delta chain is CF_1_ subunit (δ) belongs to the F-type ATPase, which utilizes the energy of a transmembrane electrochemical gradient to generate ATP by rotary catalysis [47]. F-type ATPase products would provide energy for photosynthesis carbon fixation [48]. ATP synthesis in the hydrophilic α_3_β_3_ head (CF_1_) is powered by the CF_0_ rotary motor in the membrane [49]. Previous studies have shown that the ATP synthase delta chain is mainly related to the component linkage of the F-type ATPase sector [50, 51]. In our study, ATP synthase delta chain protein was up-regulated in eCO_2_ (Fig. 3), this indicated that eCO_2_ can affect leaf redox pathways by changing the F-type ATPase subunit accumulation. Our results confirmed that ATP synthase delta chain act as a stator to prevent unproductive rotation of CF_1_ with CF_0_, this is consistent with previous study [49].

### eCO_2_ promotes up-regulation of RbcS and Rca

Rubisco is L_8_S_8_ hexadecamer complex [52] and inefficient [53]. RbcS regulates Rubisco through coordinated expression of RbcL and RbcS in plants [11]. RbcS is linked to the folded RbcL subunits assemble [54] and as a ‘reservoir’ for CO_2_ storage [17]. In our results, RbcS was up-regulated in eCO_2_ (Fig. 4), this indicated that RbcS not only has high affinity with CO_2_, but also responds to eCO_2_ in the environment. It has reported that *RbcS* mRNA levels and RbcS synthesis simultaneously increased in RbcS-sense plants [11]. The *RbcS* transcript was found to be inhibited in source of sugar (sucrose or glucose) in the media of photoautotrophic *Chenopodium* callus and some plants, but over-expression of *RbcS* was found in low CO_2_ [55]. Interestingly, *RbcS* mRNA level was up-regulated in C0, and down-regulated in Cs and CK, which indicate that the medium with sugar inhibits the expression of RbcS, this is consistent with previous studies. The amount of RbcS synthesize was tightly correlated with *RbcL* mRNA level [11]. In our research, large amounts of RbcS accumulated under eCO_2_ but there was no significant change in *RbcL* mRNA level. This result showed that RbcS accumulated not only associated with *RbcL* mRNA level, but also related to CO_2_ concentration. It has reported that long-term growth of Arabidopsis at high CO_2_ (1000 μmol·mol^− 1^) resulted in nonstructural carbohydrates increased and an even greater decline in mRNA of *RbcS* [56]. Nevertheless, the mechanism of eCO_2_ regulates RbcS accumulated would research in future.

Sugar phosphate inhibits Rubisco activity [57], such as RuBP, CATP and Xu5P [12]. Rca catalyzes the remodeling of inactive Rubisco, releases it’s bound sugar phosphate and activate Rubisco [20]. Heat [23], drought [24] and salt [25] could increase Rca. In our results, Rca was up-regulated under eCO_2_. Through the previous analysis, LHCII, PsbQ, PsbE, PsbD, PsaN and ATP synthase delta chain were up-regulated, indicating that these proteins would absorb more energy and produce more ATP, which could change ATP/ADP ratio. Rca uses the hydrolysis of ATP to facilitate the dissociation of RuBP bound as an inhibitor at the active site of uncarbamylated and inactive Rubisco [58]. Therefore, the activity of Rca was affected by ADP/ATP ratio [22]. We speculated that eCO_2_ affect Rca activity by up-regulating the expression of light-harvesting proteins and F-type ATPase, and all of those changes ultimately affect the activation of Rubisco. Galmés et al. [59] reported that Rubisco content reduced was the primary driver in the regulation of Rubisco activity to eCO_2_. At normal conditions, Rca negatively affects the Rubisco content [60]. However, Rca level is a major limiting factor of non-steady-state photosynthesis [61]. Therefore, Rca up-regulated to adjust the non-steady-state photosynthesis caused by eCO_2_. Overproduction of Rubisco does not enhance photorespiration as well as CO_2_ assimilation probably due to partial deactivation of Rubisco [62]. Rca was negatively correlated with mRNA levels, it is suggested that changes in the expression of these proteins are regulated at their translation levels by eCO_2_.

## Conclusions

The detailed analysis of transcriptome and proteome of grape (*V. vinifera* L. cv. ‘Pinot Noir’) plantlets in vitro under differential concentration of CO_2_ revealed crucial molecular mechanism difference in transformation from heterotrophic to autotrophic. The results indicated that eCO_2_ triggers the RbcS and Rca up-regulated, then promoting photosynthesis and then advancing transformation of grape plantlets from heterotrophic to autotrophic. The study provided deep refinements into the existing knowledge of plantlets in vitro response to eCO_2_, and the molecular mechanism was revealed through identification and comparative analysis of genes and proteins from photosynthesis-antenna, photosynthesis and metabolism pathways. The expression level of *RbcS* was not related to protein expression and the expression of *Rca* was highly inverse correlated with protein expression. Consequently, these datas provide clues as to the fundamental regulatory network targeted by eCO_2_, and will lead to future functional analyses that may be valuable for both agronomic improvement and our understanding of the means by which new phenotypes may arise.

## Methods

### Plant materials

‘Pinot Noir’ (*V. vinifera* L.) grape plantlets, which was kept in the Fruit Tree Physiology and Biotechnology Laboratory, College of Horticulture, Gansu Agricultural University, were used as test materials in an in vitro experiment. The grape plantlets were propagated in advance and were vigorous in growth without contamination. Each nodal segment (approximately 2.0 cm long) with two bud was cultured on modified B5 solid medium + IAA (0.1 mg·L^− 1^) (50 mL of medium was taken in 150 mL Erlenmeyer flasks). Plantlets were grown in controlled climate chamber (PQX-430D) at a day/night regime of 16 h/8 h (light/dark), an irradiance of 120 μmol·m^− 2^·s^− 1^, temperatures of 26 °C day and night. One climate chamber (PQX-430D-CO_2_) have TC-5000 (T) intelligent CO_2_ controller to regulate CO_2_ concentration. The CO_2_ concentration treatments were as follows: environmental atmospheric CO_2_ concentrations (380 ± 40 μmol·mol^− 1^); and elevated CO_2_ concentrations (1000 μmol·mol^− 1^). The grape plantlets were cultured with 2% sucrose designated as control (CK), with eCO_2_ while without sucrose as C0, with both 2% sucrose and eCO_2_ as Cs. Each treatment had three biological replicates with 15 plantlets per replicate. Plantlet leaves were harvested at 25 days after inoculation. The leaf samples were transferred immediately to liquid nitrogen and stored at − 80 °C for subsequent analysis. Different treatments were simultaneously sampled from three comparable plants used as three biological replications.

### Chlorophyll fluorescence parameters

Chlorophyll fluorescence parameters of functional leaves were measured using the IMAG-PAM fluorometer (MAXI Imaging-PAM, Walz, Effeltrich, Germany). All daytime measurements were carried out between 10:00 and 12:00 in the morning. After the dark adaptation, minimal fluorescence (F0), steady fluorescence (Fs) and maximum fluorescence (Fm) were respectively measured under light irradiation (0.1110 and 2700 μmol·m^− 2^·s^− 1^). The optimal photochemical efficiency of PSII (Fv/Fm), effective quantum yields of PSII (ΦPSII), photochemistry quenching (qP) and photosynthetic electron transport (ETR) were calculated according to previous equations [63].

### RNA isolation and library preparation for transcriptome analysis

Total RNA samples were extracted using the mirVana miRNA Isolation Kit (Ambion). The RNA samples were evaluated using the Agilent 2100 Bioanalyzer (Agilent Technologies, Santa Clara, CA, USA), with RNA Integrity Number (RIN) ≥ 7 were subjected to the subsequent analysis. The libraries were constructed using TruSeq Stranded mRNA LTSample Prep Kit (Illumina, San Diego, CA, USA) according to the manufacturer’s instructions. Then these libraries were sequenced on the Illumina sequencing platform (HiSeqTM 2500 or Illumina HiSeq X Ten) and 125 bp/150 bp paired-end reads were generated.

### Analysis of RNA-sequencing data

Raw data (raw reads) were filtered into clean reads using NGS QC Toolkit. The reads containing ploy-N and the low quality reads were removed to obtain the clean reads. Then the clean reads were mapped to reference genome sequence (http://www.genoscope.cns.fr/externe/GenomeBrowser/Vitis/) using HISTA 2. Briefly, the number of mapped reads for each transcript was normalized into a reads per kb per million reads value (RPKM) to calculate level of differential expression for each transcript. In analysis, a criterion of *P* value < 0.05 and fold change > 2 or fold change < 0.5 was used to identify DEG. Functional gene classification was performed using UniProtKB/Swiss-Prot database. GO enrichment and KEGG pathway enrichment analysis of DEGs were performed using the R programming language based on the hypergeometric distribution, respectively.

### qRT-PCR analysis

One micrograms total RNA was subjected to reverse transcription using SYBR Green PCR Master Mix (TaKaRa) Kit with gDNA Eraser (Perfect for Real Time). Real-time PCR was carried out by using SYBRs Premix Ex Taq II (TaKaRa) in ABI StepOne™ Plus Real-Time PCR System (Roche, Switzerland). All primers used for qRT-PCR were listed in Additional file 6: Table S5.

### Protein extraction

Fresh leaves (0.5 g) from each biological replicate were ground into power in liquid nitrogen and dissolved (vortex blending) with 500 μL extraction buffer (0.7 M sucrose, 0.1 M NaCl, 0.5 M Tris-HCl (pH 7.5), 50 mM EDTA and 0.2% DTT). The samples were grinded at the power of 60 Hz for 2 min. Then supplemented with extraction buffer for 1 mL and mixed and added with Tris-phenol buffer and mixed for 30 min at 4 °C. The mixtures were centrifuged at 7100 *g* for 10 min at 4 °C. Collect phenol supernatants and added for 5 volumes of 0.1 M cold ammonium acetate-methanol buffer and precipitated at − 20 °C overnight. The samples were centrifuged at 12,000 *g* for 10 min to collect precipitations. The precipitations were dried and dissolved in lysis buffer (1% DTT, 2% SDS, 10% glycerinum, 50 mM Tris-HCl (pH 6.8) for 3 h. The samples were centrifuged at 12000 g for 10 min to collect supernatants. The supernatants were centrifuged again to remove precipitations completely. The protein concentration was quantified by BCA method [64] and theprotein purity was detected by SDS-PAGE [65], 15μg proteins of each sample were separated on 12% SDS-PAGE gel.

### Protein digestion and iTRAQ labeling

Protein digestion was performed according to the FASP procedure [66]. Brifely, protein sample (100 μg) was subjected with 120 μL reducing buffer (10 mM DTT, 8 M Urea, 100 mM TEAB, pH 8.0) on 10 K ultrafiltration tube and the solution was incubated at 60 °C for 1 h. IAA was added to the solution with the final concentration of 50 mM in the dark at room temperature for 40 min. The solutions were centrifuged on the filters at 12,000 *g* for 20 min at 4 °C. Remove the supernatant and add TEAB (100 μL, 100 mM) to the solutions and centrifuged at 12,000 *g* for 20 min. Collection the filter units into new tubes, add TEAB (100 μL, 100 mM) and followed with 2 μL sequencing-grade trypsin (1 μg·μL^− 1^), incubated for digestion at 37 °C for 12 h. The collections of digested peptides were centrifuge at 12,000 *g* for 20 min. The solutions were collected and lyophilized. The lyophilized samples were resuspended in TEAB (100 μL, 50 mM) and 40 μL of each sample was transferred into new tubes for labeling. Each sample add iTRAQ label reagent (iTRAQ® Reagents-8plex kit, Sigma) following the manufacturer’s protocol (Applied Biosystems, Foster City, CA, USA). All labeled peptides were pooled together.

### RP chromatography separation

iTRAQ labeled peptides were fractionated by RP chromatography separation using the 1100 HPLC System (Agilent). RP separation was performed on the Agilent Zorbax Extend RP column (5 μm, 150 mm × 2.1 mm). Mobile phases A (2% acetonitrile in HPLC water) and B (98% acetonitrile in HPLC water) were used for RP gradient. The solvent gradient was set as follows: 0~8 min, 98% A; 8.00~8.01 min, 98%~ 95% A; 8.01~38 min, 95%~ 75% A; 38~50 min, 75~60% A; 50~50.01 min, 60~10% A; 50.01~60 min, 10% A; 60~60.01 min, 10~98% A; 60.01~65 min, 98% A. Tryptic peptides were separated at an eluent flow rate of 300 μL·min^− 1^ and monitored at 210 and 280 nm. Dried samples were harvested from 8 min to 50 min and elution buffer were collected in every minute and numbered from 1 to 10 with pipeline. The separated peptides were lyophilized for MS detection.

### Mass spectrometry analysis

All LC-MS/MS analyses were performed on a Q-Exactive mass spectrometer (Thermo, USA) equipped with a Nanospray Flex source (Thermo, USA). The peptides mixtures were loaded by a capillary C18 trap column (3 cm × 100 μm, C18, 3 μm, 150 Å) and separated by a C18 column (15 cm × 75 μm, C18, 3 μm, 120 Å) on an ChromXP Eksigent system (AB Sciex). The flow rate was 300 nL·min^− 1^ and linear gradient was 70 min (0~0.5 min, 95%~ 92% A; 0.5~48 min, 92%~ 74% A; 48~61 min, 74%~ 62% A; 61~61.1 min, 62%~ 15% A; 61.1~67 min, 15% A; 67~67.1, 15%~ 95% A; 67.1~70 min, 95% *A. mobile* phase A = 2% ACN/0.1% FA and B = 95% ACN/0.1% FA). Full MS scans were acquired in the mass range of 300–1600 m/z with a mass resolution of 70,000 and the AGC target value was set at 1,000,000. The 10 most intense peaks in MS were fragmented with higher-energy collisional dissociation (HCD) with collision energy of 30. MS/MS spectra were obtained with a resolution of 17,500 with an AGC target of 200,000 and a max injection time of 50 ms. The Q-E dynamic exclusion was set for 15.0 s and run under positive mode.

### Protein identification and function annotation

Raw data of iTRAQ-labeled proteins by was search against *V. vinifera* (Grape) genome protein database in National Center for Biotechnology Information (NCBI) using the Proteome DiscovererTM 2.2 (Thermo, USA). Database searches were performed with trypsin digestion specificity, and the cysteine alkylation was considered as parameters in the database searching. For protein quantification method, iTRAQ8-plex was selected. For protein identification, a decoy database search approach was used to determine the false discovery rate (FDR) with acceptance if their FDR < 1.0% while protein identification containing at least two peptides.

The molecular functions of the identified proteins were classified according to their gene ontology annotations and their biological functions. Only the proteins identified with at least two different peptides and *P* value < 0.05, and quantified with a ratio of fold change > 1.4 or fold change < 5/7 and *P* value < 0.05, were considered. The NCBI and Uniprot databases were chosen to the validation and annotation of the protein sequences. Gene Ontology (GO) annotation for the identified proteins was assigned according to Uniprot database (http://www.uniprot.org).

### Statistical analysis

The control and treatment groups were analyzed for statistical significance of differences between multiple groups using one-way ANOVA followed by Duncan’s multiple comparisons test. All calculations were performed using SPSS software (version 21; IBM, Armonk, NY, USA). All results are presented as mean ± SD from 3 independent biological replications. Treatment means were separated by the Duncan multiple range test at *P* value less than 0.01. We use min-max normalization method through the R programming language (3.4.3, pheatmap) to analysis transcriptional and proteomic represent expression values of heat map.

## Additional files

Additional file 1: **Table S1.** Effect of eCO_2_ on fresh weight, dry weight, leaf area and plant height. (DOC 29 kb)

Additional file 2: **Figure S1.** The protein sample analysis by SDS-PAGE. (TIF 508 kb)

Additional file 3: **Table S2.** The category with the most DEGs of Cs and C0 compare with CK. (DOC 112 kb)

Additional file 4: **Table S3.** The category with the most DEPs of Cs and C0 compare with CK. (DOC 100 kb)

Additional file 5: **Table S4.** The top KEGG pathways of Cs versus CK, C0 versus CK and Cs versus C0. (DOC 90 kb)

Additional file 6: **Table S5.** Sequences of primer employed in qRT-PCR analysis. (DOC 59 kb)

## Abbreviations

DEGs: Differentially expressed genes;
DEPs: Differentially expressed proteins
eCO_2_: Elevated CO_2_ concentration
GO: Gene ontology
iTRAQ: Isobaric Tag for Relative Absolute Quantitation
KEGG: Kyoto encyclopedia of genes and genomes
qRT-PCR: Quantitative real-time PCR
RbcS: Ribulose bisphosphate carboxylase small chain
Rca: Rubisco activase
Rubisco: Ribulose-1, 5-bisphosphate carboxylase/oxygenase
