# Supplementary material for: Elevated CO2 concentration promotes photosynthesis of grape (Vitis vinifera L. cv. ‘Pinot noir’) plantlet in vitro by regulating RbcS and Rca revealed by proteomic and transcriptomic profiles
Source: BMC Plant Biol. 2019 Jan 29;19:42. doi: 10.1186/s12870-019-1644-y (PMC6352424; doi:10.1186/s12870-019-1644-y)
Supplement: Supplementary file 1 — Table S1. Effect of eCO2 on fresh weight, dry weight, leaf area and plant height. (DOC 29 kb) [file 12870_2019_1644_MOESM1_ESM.doc]

**Table S1:** Effect of eCO_2_ on fresh weight, dry weight, leaf area and plant height

|  | Shoot FW (g) | Root FW(g) | ShootDW(g) | Root DW (g) | LA (cm^2^) | Plant height(cm) |
| --- | --- | --- | --- | --- | --- | --- |
| CK | 0.53±0.03c | 0.59±0.06a | 0.05±0.03b | 0.03±0.016a | 4.07±0.07b | 5.78±0.33b |
| C0 | 0.82±0.07b | 0.27±0.03b | 0.07±0.02b | 0.01±0.004a | 6.67±0.04a | 6.29±0.19b |
| Cs | 1.02±0.04a | 0.50±0.04a | 0.16±0.03a | 0.02±0.005a | 7.97±0.06a | 7.56±0.08a |
