# Supplementary figures and images for: Elevated CO2 concentration promotes photosynthesis of grape (Vitis vinifera L. cv. ‘Pinot noir’) plantlet in vitro by regulating RbcS and Rca revealed by proteomic and transcriptomic profiles
Source: BMC Plant Biol. 2019 Jan 29;19:42. doi: 10.1186/s12870-019-1644-y (PMC6352424; doi:10.1186/s12870-019-1644-y)

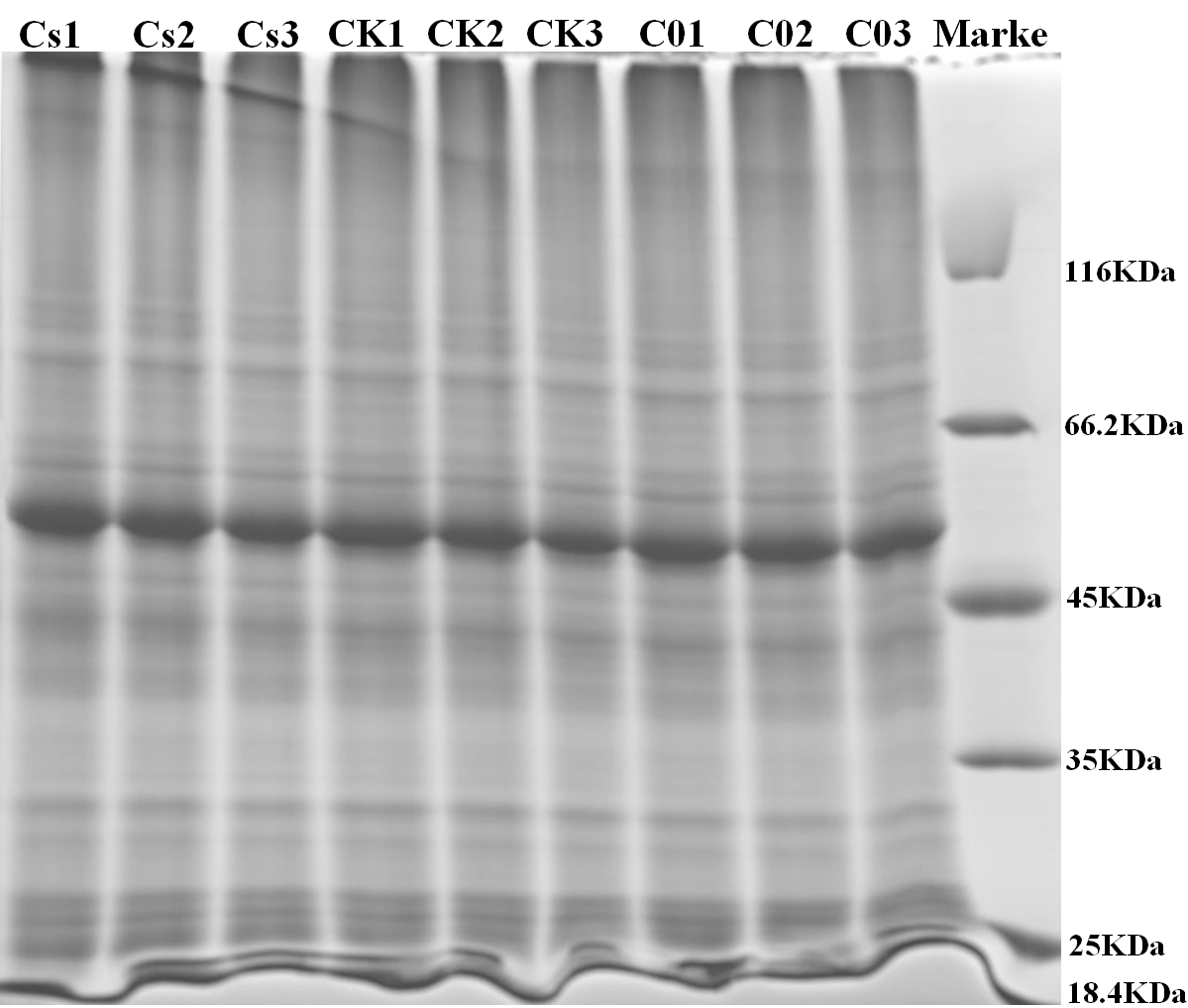

Supplement: Supplementary file 2 — Figure S1. The protein sample analysis by SDS-PAGE. (TIF 508 kb) [file 12870_2019_1644_MOESM2_ESM.tif]
