# Supplementary material for: Elevated CO2 concentration promotes photosynthesis of grape (Vitis vinifera L. cv. ‘Pinot noir’) plantlet in vitro by regulating RbcS and Rca revealed by proteomic and transcriptomic profiles
Source: BMC Plant Biol. 2019 Jan 29;19:42. doi: 10.1186/s12870-019-1644-y (PMC6352424; doi:10.1186/s12870-019-1644-y)
Supplement: Supplementary file 3 — Table S2. The category with the most DEGs of Cs and C0 compare with CK. (DOC 112 kb) [file 12870_2019_1644_MOESM3_ESM.doc]

| **Table S2 A:** The category with the most DEGs of Cs compare with CK | | | | | | | | | |
| --- | --- | --- | --- | --- | --- | --- | --- | --- | --- |
| id | term | category | ListHits | ListTotal | PopHits | PopTotal | *P* val | *P* adj | Enrichment_score |
| GO:0016021 | integral component of membrane | cellular_component | 99 | 596 | 3539 | 17750 | 0.99 | 0.99 | 0.83 |
| GO:0005737 | cytoplasm | cellular_component | 94 | 596 | 2294 | 17750 | 0.02 | 0.04 | 1.22 |
| GO:0005634 | nucleus | cellular_component | 89 | 596 | 3930 | 17750 | 0.99 | 0.99 | 0.67 |
| GO:0005886 | plasma membrane | cellular_component | 55 | 596 | 2197 | 17750 | 0.99 | 0.99 | 0.75 |
| GO:0005576 | extracellular region | cellular_component | 46 | 596 | 818 | 17750 | 0.00 | 0.00 | 1.67 |
| GO:0005618 | cell wall | cellular_component | 35 | 596 | 555 | 17750 | 0.00 | 0.00 | 1.88 |
| GO:0005829 | cytosol | cellular_component | 32 | 596 | 1421 | 17750 | 0.99 | 0.99 | 0.67 |
| GO:0048046 | apoplast | cellular_component | 26 | 596 | 437 | 17750 | 0.00 | 0.01 | 1.77 |
| GO:0009506 | plasmodesma | cellular_component | 22 | 596 | 582 | 17750 | 0.24 | 0.32 | 1.13 |
| GO:0016020 | membrane | cellular_component | 22 | 596 | 842 | 17750 | 0.87 | 0.93 | 0.78 |
| GO:0006952 | defense response | biological_process | 70 | 596 | 603 | 17751 | 0.00 | 0.00 | 3.46 |
| GO:0006351 | transcription, DNA-templated | biological_process | 59 | 596 | 1934 | 17751 | 0.76 | 0.83 | 0.91 |
| GO:0008152 | metabolic process | biological_process | 36 | 596 | 219 | 17751 | 0.00 | 0.00 | 4.90 |
| GO:0009737 | response to abscisic acid | biological_process | 25 | 596 | 315 | 17751 | 0.00 | 0.00 | 2.36 |
| GO:0009058 | biosynthetic process | biological_process | 23 | 596 | 99 | 17751 | 0.00 | 0.00 | 6.92 |
| GO:0009651 | response to salt stress | biological_process | 23 | 596 | 439 | 17751 | 0.01 | 0.04 | 1.56 |
| GO:0009734 | auxin-activated signaling pathway | biological_process | 20 | 596 | 282 | 17751 | 0.00 | 0.01 | 2.11 |
| GO:0009607 | response to biotic stimulus | biological_process | 18 | 596 | 76 | 17751 | 0.00 | 0.00 | 7.05 |
| GO:0006749 | glutathione metabolic process | biological_process | 17 | 596 | 50 | 17751 | 0.00 | 0.00 | 10.13 |
| GO:0006355 | regulation of transcription, DNA-templated | biological_process | 17 | 596 | 1300 | 17751 | 0.99 | 1 | 0.39 |
| GO:0003700 | transcription factor activity, sequence-specific DNA binding | molecular_function | 54 | 596 | 1288 | 17751 | 0.04 | 0.08 | 1.25 |
| GO:0005524 | ATP binding | molecular_function | 48 | 596 | 2118 | 17751 | 0.99 | 1 | 0.67 |
| GO:0046872 | metal ion binding | molecular_function | 47 | 596 | 1862 | 17751 | 0.98 | 1 | 0.75 |
| GO:0020037 | heme binding | molecular_function | 37 | 596 | 479 | 17751 | 0.00 | 0.00 | 2.30 |
| GO:0004364 | glutathione transferase activity | molecular_function | 35 | 596 | 99 | 17751 | 0.00 | 0.00 | 10.53 |
| GO:0005506 | iron ion binding | molecular_function | 34 | 596 | 420 | 17751 | 0.00 | 0.00 | 2.41 |
| GO:0003677 | DNA binding | molecular_function | 34 | 596 | 1418 | 17751 | 0.98 | 1 | 0.71 |
| GO:0050350 | trihydroxystilbene synthase activity | molecular_function | 23 | 596 | 36 | 17751 | 0.00 | 0.00 | 19.03 |
| GO:0043565 | sequence-specific DNA binding | molecular_function | 22 | 596 | 487 | 17751 | 0.06 | 0.12 | 1.35 |
| GO:0016491 | oxidoreductase activity | molecular_function | 19 | 596 | 299 | 17751 | 0.00 | 0.01 | 1.89 |

The category with the most DEPs of Cs compare with S1, arrange by the number of every category.

| **Table S2 B:** The category with the most DEGs of C0 compare with CK | | | | | | | | | |
| --- | --- | --- | --- | --- | --- | --- | --- | --- | --- |
| id | term | category | ListHits | ListTotal | PopHits | PopTotal | *P* val | *P* adj | Enrichment_score |
| GO:0005737 | cytoplasm | cellular_component | 98 | 572 | 2294 | 17750 | 0.00 | 0.01 | 1.33 |
| GO:0016021 | integral component of membrane | cellular_component | 97 | 572 | 3539 | 17750 | 0.96 | 0.99 | 0.85 |
| GO:0005634 | nucleus | cellular_component | 84 | 572 | 3930 | 17750 | 0.99 | 0.99 | 0.66 |
| GO:0005576 | extracellular region | cellular_component | 58 | 572 | 818 | 17750 | 0.00 | 0.00 | 2.20 |
| GO:0005886 | plasma membrane | cellular_component | 55 | 572 | 2197 | 17750 | 0.98 | 0.99 | 0.78 |
| GO:0005618 | cell wall | cellular_component | 40 | 572 | 555 | 17750 | 0.00 | 0.00 | 2.24 |
| GO:0005829 | cytosol | cellular_component | 31 | 572 | 1421 | 17750 | 0.99 | 0.99 | 0.68 |
| GO:0009507 | chloroplast | cellular_component | 26 | 572 | 1449 | 17750 | 0.99 | 0.99 | 0.56 |
| GO:0048046 | apoplast | cellular_component | 25 | 572 | 437 | 17750 | 0.00 | 0.01 | 1.78 |
| GO:0005783 | endoplasmic reticulum | cellular_component | 21 | 572 | 632 | 17750 | 0.39 | 0.46 | 1.03 |
| GO:0005737 | cytoplasm | cellular_component | 98 | 572 | 2294 | 17750 | 0.00 | 0.01 | 1.33 |
| GO:0006351 | transcription, DNA-templated | biological_process | 48 | 572 | 1934 | 17750 | 0.97 | 0.99 | 0.77 |
| GO:0006355 | regulation of transcription, DNA-templated | biological_process | 25 | 572 | 1300 | 17750 | 0.99 | 0.99 | 0.60 |
| GO:0009058 | biosynthetic process | biological_process | 21 | 572 | 99 | 17750 | 0.00 | 0.00 | 6.58 |
| GO:0008152 | metabolic process | biological_process | 21 | 572 | 219 | 17750 | 0.00 | 0.00 | 2.98 |
| GO:0009737 | response to abscisic acid | biological_process | 20 | 572 | 315 | 17750 | 0.00 | 0.01 | 1.97 |
| GO:0009651 | response to salt stress | biological_process | 19 | 572 | 439 | 17750 | 0.08 | 0.13 | 1.34 |
| GO:0009734 | auxin-activated signaling pathway | biological_process | 17 | 572 | 282 | 17750 | 0.00 | 0.02 | 1.87 |
| GO:0071555 | cell wall organization | biological_process | 16 | 572 | 331 | 17750 | 0.04 | 0.08 | 1.50 |
| GO:0046872 | metal ion binding | molecular_function | 52 | 572 | 1862 | 17750 | 0.85 | 0.90 | 0.87 |
| GO:0003700 | transcription factor activity, sequence-specific DNA binding | molecular_function | 45 | 572 | 1288 | 17750 | 0.25 | 0.33 | 1.08 |
| GO:0005524 | ATP binding | molecular_function | 36 | 572 | 2118 | 17750 | 0.99 | 0.99 | 0.53 |
| GO:0003677 | DNA binding | molecular_function | 31 | 572 | 1418 | 17750 | 0.99 | 0.99 | 0.68 |
| GO:0020037 | heme binding | molecular_function | 27 | 572 | 479 | 17750 | 0.00 | 0.01 | 1.75 |
| GO:0004364 | glutathione transferase activity | molecular_function | 25 | 572 | 99 | 17750 | 0.00 | 0.00 | 7.84 |
| GO:0005506 | iron ion binding | molecular_function | 25 | 572 | 420 | 17750 | 0.00 | 0.01 | 1.85 |
| GO:0008270 | zinc ion binding | molecular_function | 24 | 572 | 872 | 17750 | 0.76 | 0.81 | 0.85 |
| GO:0016491 | oxidoreductase activity | molecular_function | 22 | 572 | 299 | 17750 | 0.00 | 0.00 | 2.28 |
| GO:0043565 | sequence-specific DNA binding | molecular_function | 21 | 572 | 487 | 17750 | 0.07 | 0.13 | 1.34 |

The category with the most DEPs of C1 compare with CK, arrange by the number of every category.
