# Supplementary material for: Elevated CO2 concentration promotes photosynthesis of grape (Vitis vinifera L. cv. ‘Pinot noir’) plantlet in vitro by regulating RbcS and Rca revealed by proteomic and transcriptomic profiles
Source: BMC Plant Biol. 2019 Jan 29;19:42. doi: 10.1186/s12870-019-1644-y (PMC6352424; doi:10.1186/s12870-019-1644-y)
Supplement: Supplementary file 4 — Table S3. The category with the most DEPs of Cs and C0 compare with CK. (DOC 100 kb) [file 12870_2019_1644_MOESM4_ESM.doc]

| **Table S3 A:** The category with the most DEPs of Cs compare with CK | | | | | | | |
| --- | --- | --- | --- | --- | --- | --- | --- |
| maxLevel | levels | GO_Name | GO_ID | category | Count | Pop_Hit | Pvalue |
| 2 | 2 | cell part | GO:0044464 | cellular_component | 68 | 11627 | 1.55E-04 |
| 2 | 2 | cell | GO:0005623 | cellular_component | 68 | 11702 | 2.15E-04 |
| 3 | 3 | intracellular | GO:0005622 | cellular_component | 66 | 9889 | 5.70E-07 |
| 3 | 3 | intracellular part | GO:0044424 | cellular_component | 65 | 9502 | 3.26E-07 |
| 4 | 4 | cytoplasm | GO:0005737 | cellular_component | 56 | 6085 | 1.60E-10 |
| 4 | 3,4 | intracellular organelle | GO:0043229 | cellular_component | 55 | 7809 | 1.86E-05 |
| 2 | 2 | organelle | GO:0043226 | cellular_component | 55 | 7875 | 2.54E-05 |
| 5 | 4,5 | cytoplasmic part | GO:0044444 | cellular_component | 53 | 4723 | 2.87E-13 |
| 5 | 4,5 | intracellular membrane-bounded organelle | GO:0043231 | cellular_component | 52 | 7212 | 2.60E-05 |
| 3 | 3 | membrane-bounded organelle | GO:0043227 | cellular_component | 52 | 7289 | 3.73E-05 |
| 2 | 2 | metabolic process | GO:0008152 | biological_process | 81 | 14308 | 2.02E-01 |
| 2 | 2 | cellular process | GO:0009987 | biological_process | 68 | 13460 | 8.89E-01 |
| 3 | 3 | cellular metabolic process | GO:0044237 | biological_process | 62 | 10235 | 9.69E-02 |
| 3 | 3 | organic substance metabolic process | GO:0071704 | biological_process | 61 | 10889 | 3.61E-01 |
| 2 | 2 | single-organism process | GO:0044699 | biological_process | 54 | 10052 | 5.64E-01 |
| 3 | 3 | primary metabolic process | GO:0044238 | biological_process | 51 | 10306 | 8.56E-01 |
| 3 | 3 | single-organism metabolic process | GO:0044710 | biological_process | 48 | 5413 | 4.56E-05 |
| 3 | 3 | nitrogen compound metabolic process | GO:0006807 | biological_process | 43 | 8654 | 8.06E-01 |
| 3 | 3 | biosynthetic process | GO:0009058 | biological_process | 42 | 5469 | 4.79E-03 |
| 4 | 4 | cellular biosynthetic process | GO:0044249 | biological_process | 38 | 4858 | 5.99E-03 |
| 2 | 2 | catalytic activity | GO:0003824 | molecular_function | 59 | 10610 | 4.81E-01 |
| 2 | 2 | binding | GO:0005488 | molecular_function | 57 | 10756 | 7.11E-01 |
| 3 | 3 | heterocyclic compound binding | GO:1901363 | molecular_function | 43 | 7467 | 3.74E-01 |
| 3 | 3 | organic cyclic compound binding | GO:0097159 | molecular_function | 43 | 7469 | 3.75E-01 |
| 3 | 3 | ion binding | GO:0043167 | molecular_function | 34 | 6580 | 7.10E-01 |
| 5 | 5 | metal ion binding | GO:0046872 | molecular_function | 25 | 3294 | 4.99E-02 |
| 4 | 4 | cation binding | GO:0043169 | molecular_function | 25 | 3317 | 5.35E-02 |
| 3 | 3 | oxidoreductase activity | GO:0016491 | molecular_function | 21 | 1974 | 2.04E-03 |
| 3 | 3 | transferase activity | GO:0016740 | molecular_function | 21 | 4019 | 6.42E-01 |
| 4 | 4 | tetrapyrrole binding | GO:0046906 | molecular_function | 1 | 1 | 5.49E-03 |

| **Table S3 B:** The category with the most DEPs of C0 compare with CK | | | | | | | |
| --- | --- | --- | --- | --- | --- | --- | --- |
| maxLevel | levels | GO_Name | GO_ID | category | Count | Pop_Hit | Pvalue |
| 2 | 2 | cell part | GO:0044464 | cellular_component | 36 | 11627 | 3.34E-02 |
| 2 | 2 | cell | GO:0005623 | cellular_component | 36 | 11702 | 3.87E-02 |
| 3 | 3 | intracellular part | GO:0044424 | cellular_component | 35 | 9502 | 6.54E-04 |
| 3 | 3 | intracellular | GO:0005622 | cellular_component | 35 | 9889 | 1.85E-03 |
| 4 | 4 | cytoplasm | GO:0005737 | cellular_component | 32 | 6085 | 4.22E-07 |
| 4 | 3,4 | intracellular organelle | GO:0043229 | cellular_component | 29 | 7809 | 6.10E-03 |
| 2 | 2 | organelle | GO:0043226 | cellular_component | 29 | 7875 | 7.10E-03 |
| 5 | 4,5 | cytoplasmic part | GO:0044444 | cellular_component | 28 | 4723 | 6.06E-07 |
| 5 | 4,5 | intracellular membrane-bounded organelle | GO:0043231 | cellular_component | 27 | 7212 | 9.26E-03 |
| 3 | 3 | membrane-bounded organelle | GO:0043227 | cellular_component | 27 | 7289 | 1.10E-02 |
| 2 | 2 | metabolic process | GO:0009768 | biological_process | 45 | 14308 | 2.66E-01 |
| 2 | 2 | cellular process | GO:0009765 | biological_process | 35 | 13460 | 9.60E-01 |
| 3 | 3 | cellular metabolic process | GO:0018298 | biological_process | 31 | 10235 | 5.03E-01 |
| 3 | 3 | organic substance metabolic process | GO:0019684 | biological_process | 30 | 10889 | 7.98E-01 |
| 3 | 3 | primary metabolic process | GO:0015979 | biological_process | 29 | 10306 | 7.33E-01 |
| 2 | 2 | single-organism process | GO:0006091 | biological_process | 25 | 10052 | 9.34E-01 |
| 3 | 3 | nitrogen compound metabolic process | GO:0009628 | biological_process | 23 | 8654 | 8.17E-01 |
| 4 | 4 | organonitrogen compound metabolic process | GO:0009416 | biological_process | 22 | 5279 | 4.41E-02 |
| 3 | 3 | single-organism metabolic process | GO:0009314 | biological_process | 22 | 5413 | 5.70E-02 |
| 2 | 2 | response to stimulus | GO:0046394 | biological_process | 21 | 4008 | 3.83E-03 |
| 2 | 2 | catalytic activity | GO:0003824 | molecular_function | 28 | 10610 | 8.67E-01 |
| 2 | 2 | binding | GO:0005488 | molecular_function | 25 | 10756 | 9.82E-01 |
| 3 | 3 | heterocyclic compound binding | GO:1901363 | molecular_function | 19 | 7467 | 8.43E-01 |
| 3 | 3 | organic cyclic compound binding | GO:0097159 | molecular_function | 19 | 7469 | 8.44E-01 |
| 3 | 3 | ion binding | GO:0043167 | molecular_function | 17 | 6580 | 8.01E-01 |
| 5 | 5 | metal ion binding | GO:0046872 | molecular_function | 15 | 3294 | 4.88E-02 |
| 4 | 4 | cation binding | GO:0043169 | molecular_function | 15 | 3317 | 5.15E-02 |
| 4 | 4 | tetrapyrrole binding | GO:0046906 | molecular_function | 11 | 576 | 7.14E-07 |
| 3 | 3 | hydrolase activity | GO:0016787 | molecular_function | 11 | 3772 | 5.73E-01 |
| 3 | 3 | pigment binding | GO:0031409 | molecular_function | 1 | 4 | 1.18E-02 |
