# Supplementary material for: Elevated CO2 concentration promotes photosynthesis of grape (Vitis vinifera L. cv. ‘Pinot noir’) plantlet in vitro by regulating RbcS and Rca revealed by proteomic and transcriptomic profiles
Source: BMC Plant Biol. 2019 Jan 29;19:42. doi: 10.1186/s12870-019-1644-y (PMC6352424; doi:10.1186/s12870-019-1644-y)
Supplement: Supplementary file 5 — Table S4. The top KEGG pathways of Cs versus CK, C0 versus CK and Cs versus C0. (DOC 90 kb) [file 12870_2019_1644_MOESM5_ESM.doc]

**Table S4:** The top KEGG pathways of Cs versus CK, C0 versus CK and Cs versus C0

**A :****Cs versus CK**

| **Pathway Name** | **Pathway ID** | **Pvalue** | **Pvalue**  **adjusted** | **Count** | **Pop Hit** | **List**  **Total** | **Background Genes** |
| --- | --- | --- | --- | --- | --- | --- | --- |
| Photosynthesis - antenna proteins | vvi00196 | 8.19E-12 | 4.26E-10 | 8 | 19 | 54 | 4941 |
| Metabolic pathways | vvi01100 | 5.73E-10 | 1.49E-08 | 44 | 1992 | 54 | 4941 |
| Biosynthesis of secondary metabolites | vvi01110 | 6.74E-05 | 8.91E-04 | 26 | 1168 | 54 | 4941 |
| Carbon metabolism | vvi01200 | 6.85E-05 | 8.91E-04 | 11 | 253 | 54 | 4941 |
| Biosynthesis of amino acids | vvi01230 | 1.33E-04 | 1.39E-03 | 10 | 226 | 54 | 4941 |
| Valine, leucine and isoleucine biosynthesis | vvi00290 | 1.06E-03 | 9.16E-03 | 3 | 19 | 54 | 4941 |
| Glycine, serine and threonine metabolism | vvi00260 | 6.19E-03 | 4.60E-02 | 4 | 68 | 54 | 4941 |
| Glyoxylate and dicarboxylate metabolism | vvi00630 | 7.57E-03 | 4.92E-02 | 4 | 72 | 54 | 4941 |
| Photosynthesis | vvi00195 | 1.24E-02 | 7.15E-02 | 4 | 83 | 54 | 4941 |
| 2-Oxocarboxylic acid metabolism | vvi01210 | 2.84E-02 | 1.46E-01 | 3 | 61 | 54 | 4941 |
| Carbon fixation in photosynthetic organisms | vvi00710 | 3.09E-02 | 1.46E-01 | 3 | 63 | 54 | 4941 |
| Pantothenate and CoA biosynthesis | vvi00770 | 3.70E-02 | 1.60E-01 | 2 | 28 | 54 | 4941 |

**B**: C0 versus CK

| **Pathway Name** | **Pathway ID** | **Pvalue** | **Pvalue**  **adjusted** | **Count** | **Pop Hit** | **List**  **Total** | **Background Genes** |
| --- | --- | --- | --- | --- | --- | --- | --- |
| Photosynthesis - antenna proteins | vvi00196 | 2.58E-14 | 5.15E-13 | 8 | 19 | 28 | 4941 |
| Photosynthesis | vvi00195 | 8.60E-05 | 8.60E-04 | 5 | 83 | 28 | 4941 |
| Metabolic pathways | vvi01100 | 2.01E-04 | 1.34E-03 | 21 | 1992 | 28 | 4941 |
| Phenylpropanoid biosynthesis | vvi00940 | 2.25E-02 | 1.13E-01 | 4 | 194 | 28 | 4941 |
| Phenylalanine, tyrosine and tryptophan biosynthesis | vvi00400 | 3.21E-02 | 1.28E-01 | 2 | 50 | 28 | 4941 |
| Circadian rhythm - plant | vvi04712 | 5.90E-02 | 1.97E-01 | 2 | 70 | 28 | 4941 |
| Flavonoid biosynthesis | vvi00941 | 7.79E-02 | 2.23E-01 | 2 | 82 | 28 | 4941 |
| Linoleic acid metabolism | vvi00591 | 9.74E-02 | 2.28E-01 | 1 | 18 | 28 | 4941 |
| Biosynthesis of secondary metabolites | vvi01110 | 1.03E-01 | 2.28E-01 | 10 | 1168 | 28 | 4941 |
| Ascorbate and aldarate metabolism | vvi00053 | 1.99E-01 | 3.99E-01 | 1 | 39 | 28 | 4941 |
| Protein export | vvi03060 | 2.44E-01 | 4.44E-01 | 1 | 49 | 28 | 4941 |
| alpha-Linolenic acid metabolism | vvi00592 | 2.99E-01 | 4.44E-01 | 1 | 62 | 28 | 4941 |
| Phenylalanine metabolism | vvi00360 | 3.03E-01 | 4.44E-01 | 1 | 63 | 28 | 4941 |
| Cyanoamino acid metabolism | vvi00460 | 3.11E-01 | 4.44E-01 | 1 | 65 | 28 | 4941 |
| Peroxisome | vvi04146 | 3.45E-01 | 4.60E-01 | 1 | 74 | 28 | 4941 |
| Biosynthesis of amino acids | vvi01230 | 3.69E-01 | 4.61E-01 | 2 | 226 | 28 | 4941 |

**C:** Cs versus C0

| Photosynthesis - antenna proteins | **vvi00196** | **3.79E-05** | **9.87E-04** | **3** | **19** | **18** | **4941** |
| --- | --- | --- | --- | --- | --- | --- | --- |
| Cysteine and methionine metabolism | vvi00270 | 4.69E-03 | 5.33E-02 | 3 | 96 | 18 | 4941 |
| Metabolic pathways | vvi01100 | 6.14E-03 | 5.33E-02 | 13 | 1992 | 18 | 4941 |
| Circadian rhythm - plant | vvi04712 | 2.62E-02 | 1.50E-01 | 2 | 70 | 18 | 4941 |
| Monobactam biosynthesis | vvi00261 | 2.88E-02 | 1.50E-01 | 1 | 8 | 18 | 4941 |
| Flavonoid biosynthesis | vvi00941 | 3.51E-02 | 1.52E-01 | 2 | 82 | 18 | 4941 |
| Glycosphingolipid biosynthesis - globo series | vvi00603 | 4.29E-02 | 1.59E-01 | 1 | 12 | 18 | 4941 |
| Lysine biosynthesis | vvi00300 | 4.99E-02 | 1.62E-01 | 1 | 14 | 18 | 4941 |
| Selenocompound metabolism | vvi00450 | 5.68E-02 | 1.64E-01 | 1 | 16 | 18 | 4941 |
| Sphingolipid metabolism | vvi00600 | 9.74E-02 | 2.53E-01 | 1 | 28 | 18 | 4941 |
| Sulfur metabolism | vvi00920 | 1.17E-01 | 2.54E-01 | 1 | 34 | 18 | 4941 |
| Carotenoid biosynthesis | vvi00906 | 1.17E-01 | 2.54E-01 | 1 | 34 | 18 | 4941 |
| Ascorbate and aldarate metabolism | vvi00053 | 1.33E-01 | 2.66E-01 | 1 | 39 | 18 | 4941 |
| Galactose metabolism | vvi00052 | 1.95E-01 | 2.95E-01 | 1 | 59 | 18 | 4941 |
| Biosynthesis of amino acids | vvi01230 | 1.98E-01 | 2.95E-01 | 2 | 226 | 18 | 4941 |
| 2-Oxocarboxylic acid metabolism | vvi01210 | 2.01E-01 | 2.95E-01 | 1 | 61 | 18 | 4941 |
| Carbon fixation in photosynthetic organisms | vvi00710 | 2.07E-01 | 2.95E-01 | 1 | 63 | 18 | 4941 |
| Glycerolipid metabolism | vvi00561 | 2.18E-01 | 2.95E-01 | 1 | 67 | 18 | 4941 |
| Glycine, serine and threonine metabolism | vvi00260 | 2.21E-01 | 2.95E-01 | 1 | 68 | 18 | 4941 |
| Biosynthesis of secondary metabolites | vvi01110 | 2.37E-01 | 2.95E-01 | 6 | 1168 | 18 | 4941 |
| Peroxisome | vvi04146 | 2.38E-01 | 2.95E-01 | 1 | 74 | 18 | 4941 |
| Photosynthesis | vvi00195 | 2.63E-01 | 3.11E-01 | 1 | 83 | 18 | 4941 |
| Oxidative phosphorylation | vvi00190 | 4.29E-01 | 4.84E-01 | 1 | 151 | 18 | 4941 |
