# Supplementary material for: Elevated CO2 concentration promotes photosynthesis of grape (Vitis vinifera L. cv. ‘Pinot noir’) plantlet in vitro by regulating RbcS and Rca revealed by proteomic and transcriptomic profiles
Source: BMC Plant Biol. 2019 Jan 29;19:42. doi: 10.1186/s12870-019-1644-y (PMC6352424; doi:10.1186/s12870-019-1644-y)
Supplement: Supplementary file 6 — Table S5. Sequences of primer employed in qRT-PCR analysis. (DOC 59 kb) [file 12870_2019_1644_MOESM6_ESM.doc]

| **Table S5:** Sequences of primer employed in qRT-PCR analysis | | |
| --- | --- | --- |
| Gene ID | Protein name | Primer sequence (5' to 3') |
| LOC100241887 | Lhcb6 | Forward: AACTTCTGCTGCTGTGTTGAATG |
|  |  | Reverse: CGACGACAATGAGCCTCCTG |
| LOC100252004 | Lhcb3 | Forward: AACCTTGCTGACCACCTTGC |
|  |  | Reverse: AACCGACTATGTAAGCCAACTCC |
| LOC100254533 | Lhcb2 | Forward: GATTCGTTGAAGGATACCGTGTTG |
|  |  | Reverse: CCAGGCGACCGTTCTTGATC |
| LOC100232927 | Lhcb3 | Forward: CAGCTTCTACTCCACCAGTCAAG |
|  |  | Reverse: AGTAAGTAAGGCTCTGCTTGGATG |
| LOC100251653 | Lhcb3 | Forward: GCTCAGACACCTTCATACCTCAC |
|  |  | Reverse: TCCGGCATTGTTGGCACTTG |
| LOC100241745 | Lhcb1 | Forward: CCTTCGCCAAGAACCGTGAG |
|  |  | Reverse: TTCACCGAACTTGACTCCATTCC |
| LOC100260599 | Lhcb3 | Forward: CAGTCCTCGGCTTCCAAGTTG |
|  |  | Reverse: GGTACAGGTTGTTGCCATCTCC |
| LOC100245214 | Lhcb1 | Forward: CCTCGTCTTCAATCTGTCCTTCC |
|  |  | Reverse: GCCAGTCTTCCGCATAGTCAC |
| LOC100250504 | Lhcb1 | Forward: CATCCTACTTGACTGGTGAGTTCC |
|  |  | Reverse: CGATTCTTGGCGAAGGTCTCC |
| PsbE | PsbE | Forward: ATGTCTGGAAGCACGGGAGA |
|  |  | Reverse: CCGAGGGCTCCCAAACCTA |
| PsbD | PsbD | Forward: TCGTTGGTGTCAATTAGGTGGT |
|  |  | Reverse: ACAGCGATTGGAGCAGAGAATG |
| LOC100240959 | PsbQ | Forward: TCCAGTTGAGCCTGAGACTAGC |
|  |  | Reverse:GAGACTTACGCAATCCACCAGAG |
| LOC100248911 | PetE | Forward: ACACTCACTCAGAGACAGTTGAAG |
|  |  | Reverse: GGTGCGGTGATGGTCTTGAC |
| LOC100251062 | ATP synthase delta chain | Forward: CGCCAAGAATGTGAGAATCAAGAC |
|  |  | Reverse: GAACCGCCACTTCCATACCTG |
| LOC100232935 | PsaN | Forward: CTGCCTCCTCTTCTGCCAATG |
|  |  | Reverse: TTGCTCCGCTTGTAGCCAAC |
| LOC100267942 | RbcS | Forward: ACAAGGCAGTGGTGGTGTTAGC |
|  |  | Reverse: GACGGAGGTGATGTCGGTGTTG |
| LOC100266698 | Rca | Forward: CTTCAGTTCCAAGCTCAGCCTTCC |
|  |  | Reverse: TCGTCGTCCACTTCTGCAACAAC |
| LOC100246457 | Lhcb5 | Forward: GGCTGCACTGCTTAAGGTGAAGG |
|  |  | Reverse: GCAGTTCCAGCGATAACAGTGAGC |
